# Genome-wide screen of otosclerosis in population biobanks: 27 loci and shared associations with skeletal structure

**DOI:** 10.1038/s41467-022-32936-3

**Published:** 2023-01-18

**Authors:** Joel T. Rämö, Tuomo Kiiskinen, Richard Seist, Kristi Krebs, Masahiro Kanai, Juha Karjalainen, Mitja Kurki, Eija Hämäläinen, Paavo Häppölä, Aki S. Havulinna, Heidi Hautakangas, FinnGen, Reedik Mägi, Priit Palta, Tõnu Esko, Andres Metspalu, Matti Pirinen, Konrad J. Karczewski, Samuli Ripatti, Lili Milani, Konstantina M. Stankovic, Antti Mäkitie, Mark J. Daly, Aarno Palotie

**Affiliations:** 1 Institute for Molecular Medicine Finland (FIMM), Helsinki Institute of Life Science (HiLIFE), University of Helsinki, Helsinki, Finland; 2 Department of Otolaryngology – Head and Neck Surgery, Stanford University School of Medicine, Stanford, CA USA; 3 Estonian Genome Centre, Institute of Genomics, University of Tartu, Tartu, Estonia; 4 Program in Medical and Population Genetics, Broad Institute of Harvard and MIT, Cambridge, MA USA; 5 Stanley Center for Psychiatric Research, Broad Institute of Harvard and MIT, Cambridge, MA USA; 6 Analytic and Translational Genetics Unit, Massachusetts General Hospital, Boston, MA USA; 7 Finnish Institute for Health and Welfare, Helsinki, Finland; 8 Department of Public Health, Clinicum, Faculty of Medicine, University of Helsinki, Helsinki, Finland; 9 Department of Mathematics and Statistics, Faculty of Science, University of Helsinki, Helsinki, Finland; 10 Department of Otorhinolaryngology-Head and Neck Surgery, University of Helsinki and HUS Helsinki University Hospital, Helsinki, Finland; 11 Center for Genomic Medicine, Massachusetts General Hospital, Boston, MA USA

**Keywords:** Genetics research, Molecular medicine, Disease genetics, Metabolic bone disease

## Abstract

Otosclerosis is one of the most common causes of conductive hearing loss, affecting 0.3% of the population. It typically presents in adulthood and half of the patients have a positive family history. The pathophysiology of otosclerosis is poorly understood. A previous genome-wide association study (GWAS) identified a single association locus in an intronic region of *RELN*. Here, we report a meta-analysis of GWAS studies of otosclerosis in three population-based biobanks comprising 3504 cases and 861,198 controls. We identify 23 novel risk loci (*p* < 5 × 10^−8^) and report an association in *RELN* and three previously reported candidate gene or linkage regions (*TGFB1*, *MEPE*, and OTSC7). We demonstrate developmental stage-dependent immunostaining patterns of MEPE and RUNX2 in mouse otic capsules. In most association loci, the nearest protein-coding genes are implicated in bone remodelling, mineralization or severe skeletal disorders. We highlight multiple genes involved in transforming growth factor beta signalling for follow-up studies.

## Abstract (web summary)

Otosclerosis is a common form of hearing loss, with an unclear genetic etiology. Here, the authors perform a genome-wide association study meta-analysis of otosclerosis identifying 27 genetic loci, pointing to genes involved in bone remodeling, skeletal disorders and transforming growth factor β signaling.

## Introduction

Otosclerosis is an exclusively human disorder characterized by pathologic remodeling of the bone encasing the inner ear, called the otic capsule, and is one of the most common causes of conductive hearing loss^1,2^. Hearing begins when sound-induced vibrations of the tympanic membrane and ossicles within the middle ear are transmitted via stapes footplate within the oval window to sensory cells of the inner ear. In classic otosclerosis, the conduction of sound through the ossicular chain is impeded due to fixation of the stapes footplate by pathologic bone remodeling, leading to conductive hearing loss. When bone remodeling progresses to involve the cochlear endosteum, otosclerosis can cause additional sensorineural hearing loss in 20–30% of patients, which reflects damage to the delicate intracochlear cells. Although genetic, viral, immunologic and vascular factors have been implicated, the pathogenesis of otosclerosis remains poorly understood. Therapeutic options include hearing aids which provide amplification, stapedotomy surgery which replaces the fixed stapes bone with a mobile prosthesis, and cochlear implant surgery which facilitates direct electrical stimulation of the auditory nerve in most advanced cases of otosclerosis. However, robust pharmacological or genetic therapies for otosclerosis do not yet exist.

Clinical otosclerosis has an estimated prevalence of 0.30–0.38% in populations of European descent^3^. Histologic otosclerosis without clinical symptoms is more frequent, with bony overgrowth observed in as many as 2.5% of temporal bone autopsy specimens^4^. Symptomatic otosclerosis most frequently occurs in working-age individuals between the second and fifth decades^5,6^. Initial manifestation is often limited to one ear, but eventual bilateral disease is observed in 70–80% of cases^5,6^.

Otosclerosis is highly familial, with a positive family history reported for 50–60% of cases^7,8^. Based on segregation patterns, early studies classified otosclerosis as an autosomal dominant disease with reduced penetrance^8–10^. However, efforts to identify causative genes have produced inconsistent results, with insufficient evidence for most candidate genes^11,12^. Individual targeted next-generation sequencing studies have identified potential susceptibility variants in *SERPINF1*, *MEPE*, *ACAN* and *FOXL1*, although a follow-up study of *SERPINF1* did not replicate the signal^12–16^. A genome-wide association study (GWAS) including a total of 1149 otosclerosis patients identified several intronic variants within the gene encoding Reelin (RELN) that were associated with otosclerosis in European populations^17^. Although expressed in the inner ear, the molecular mechanism by which Reelin affects otosclerosis susceptibility is unknown.

Here, we report to our knowledge the largest GWAS of otosclerosis including a total of 3504 cases and 861,198 controls from three population-based sample collections: the Finnish FinnGen study, the Estonian Biobank (EstBB), and the UK Biobank (UKBB). We identify 23 novel GWAS loci and report associations in *RELN*, in two previously reported candidate genes (*TGFB1* and *MEPE*) and in the OTSC7 linkage region^17–22^. Several discovered loci harbor genes involved in the regulation of osteoblast or osteoclast function or biomineralization. Genomic risk loci for otosclerosis also overlap with risk loci for rare skeletal disorders.

## Results

### Identification of otosclerosis cases based on electronic health records

Based on International Classification of Diseases (ICD) diagnosis codes (versions 8, 9, and 10), we identified a total of 3504 otosclerosis cases in the three biobanks, including 1563 cases in FinnGen, 985 in EstBB and 956 in UKBB, for cohort prevalences of 0.62%, 0.50%, and 0.23%, respectively (Table 1). A total of 861,198 individuals had no ICD-based diagnosis of otosclerosis and were assigned control status. A proportion of otosclerosis patients undergo stapes procedures, which reflect disease severity and the validity of the ICD-based diagnoses. Stapes procedures were registered for 616 (39.4%) otosclerosis cases in FinnGen and for 218 (22.1%) otosclerosis cases in EstBB. In UKBB, reflecting the ascertainment of a large proportion of cases based on procedural history, stapes procedures were registered for 73.2% of cases. The gender and age distribution of controls and cases in each cohort is presented in Supplementary Data 1.

**Table 1:** Identification of otosclerosis cases in the study cohorts based on ICD codes, procedure codes and self-reported data

Classification	Code(s)	Definition	FinnGen	EstBB	UKBB
ICD-10	H80	Otosclerosis (any)	—	328	—
ICD-10	H80.0	.. Involving oval window, nonobliterative	740	362	10
ICD-10	H80.1	.. Involving oval window, obliterative	223	90	3
ICD-10	H80.2	.. Cochlear	55	49	3
ICD-10	H80.8	.. Other	146	139	6
ICD-10	H80.9	.. Unspecified	742	512	304
ICD-9	387	Otosclerosis (any)	306	—	36
ICD-8	386	Otosclerosis (any)	313	—	—
Phecode	383	Otosclerosis (any)	1563	985	353
Self-report		Otosclerosis (any)	—	—	203
Self-report		Stapes procedure (any)	—	—	266
Procedure codes	DDA00, 61006	Stapedotomy	517	218	—
Procedure codes	DDB00, D171, D172	Stapedectomy	129	—	464
Any stapes procedure		Self-report or procedure code -based	616	218	700
Total cases			**1563**	**985**	**956**
Total controls			**249281**	**196516**	**415401**
Prevalence (%)			0.62	0.50	0.23

Stapes procedures were identified based on the Nomesco codes DDA00 (stapedotomy) and DDB00 (stapedectomy) in FinnGen, based on the national health insurance treatment service code 61006 (stapedotomy) in EstBB, and based on the self-reported stapedectomy operation codes (code 20004) and the OPCS4 codes D171 (stapedectomy) and D172 (revision of stapedectomy) in UKBB.

### Genomic loci associated with otosclerosis in each cohort

In case-control GWASs within the individual cohorts, we observed eleven loci associated with otosclerosis at genome-wide significance (*p* < 5×10^−8^) in FinnGen, two in UKBB and one in EstBB (Supplementary Fig. 1; Supplementary Data 2-4). One locus on chromosome 11 was associated in both FinnGen and UKBB at genome-wide significance, tagged by the lead variants rs11601767 (closest protein coding gene: *LTBP3*) and rs17676161 (*SCYL1*), respectively. The only genome-wide significant variant in EstBB was rare (MAF 0.001%) and was not included in FinnGen or UKBB.

Single-nucleotide polymorphism (SNP) based heritability, calculated from the summary statistics, was higher in FinnGen (h^2^ range on the liability scale = 0.28–0.69) than in EstBB (range = −0.09–0.23) or UKBB (range = 0.10–0.21) (Supplementary Data 5 and Supplementary Fig. 2). Attenuation ratio statistics calculated using cross-trait LD Score Regression (LDSC) were 0.36 (SE 0.11) for FinnGen, 2.36 (1.14) for EstBB and <0 for UKBB, respectively (Supplementary Data 6)^23,24^. We calculated pairwise genetic correlation r_*g*_ with LDSC using summary statistics from the different cohorts. R_*g*_ between FinnGen and UKBB was 0.70, indicating shared genetic etiology between the phenotype definitions^23^. The calculation of r_*g*_ with LDSC was not feasible for EstBB due to a negative heritability estimate with LDSC (observed scale h^2^ = −0.002 [S.E. 0.0023]), likely reflecting modest polygenic signal in the cohort. However, as the effect estimates for lead variants were largely concordant between the three cohorts (Supplementary Data 2-5), we proceeded with a fixed-effects meta-analysis.

### Twenty seven significant loci in the meta-analysis of summary statistics

In the genome-wide meta-analysis of all three cohorts (including a total of 3504 cases and 861,198 controls) we identified 1452 variants associated with otosclerosis at the genome-wide significance level (*p* < 5×10^−8^). The genomic inflation factor (λ_GC_) calculated from the meta-analysis *p*-values was 1.046, while the univariate LD Score regression intercept was closer to 1.0 at 1.02 (S.E. 0.01), indicating that part of the elevation in λ_GC_ reflected true additive polygenic effects and not spurious associations from population stratification or cryptic relatedness^24^. We used several methods to estimate the heritability of otosclerosis based on genome-wide meta-analysis summary statistics. The SNP-based heritability of otosclerosis was 0.15 (SD = 0.03) when estimated with LDSC, 0.23 (SD = 0.01) when estimated with LDAK-THIN and 0.27 (0.03) when estimated with BLD-LDAK (Supplementary Data 5).

**Fig. 1:**
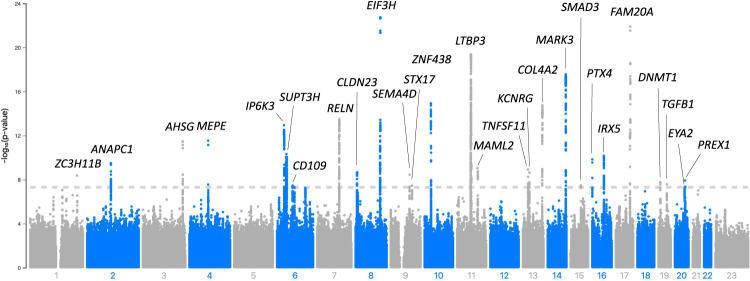
Meta-analysis of the genome-wide association studies of otosclerosis in FinnGen, EstBB and UKBB. GWAS for each individual study cohort was performed using a generalized mixed model with the saddlepoint approximation using SAIGE v0.20, using a kinship matrix as a random effect and covariates as fixed effects. Results are presented for a fixed-effect meta-analysis of effect estimates from the three cohorts, including a total of 3504 cases and 861,198 controls. In the Manhattan plot, chromosomal positions are indicated on the x-axis and -log_10_(*p*-value) is presented on the y-axis for each variant. Twenty-seven loci reached genome-wide significance (two-sided *p*-value < 5×10^−8^ to account for multiple comparisons, marked by the dashed line). The included variants were present in at least two cohorts with a cross-cohort minor allele frequency > 0.1% and imputation INFO score ≥ 0.7. The loci are annotated by the names of the coding genes nearest to the lead variants. The exact *p*-values corresponding to lead variants in each locus are presented in Table 2. Blue and gray colours are used to visually separate chromosomes.

The significant variants in the meta-analysis clustered in a total of 27 loci (regions at least 1.5MB apart) (Fig. 1; Table 2; Supplementary Data 7; Supplementary Fig. 3), including the previously reported *RELN* locus and 26 loci not previously reported in a GWAS study of otosclerosis^17^. All but one of the lead variants in the 27 loci were common with a cross-cohort effect allele frequency (EAF) of at least 11% (Table 2). We also report suggestive-level loci (*p* < 1×10^−6^) not reaching genome-wide significance in Supplementary Data 8.

**Table 2:** Lead variants for 27 significant association loci in the genome-wide association study meta-analysis of otosclerosis

Rsid	Chr	Position	Effect allele	Non-effect allele	EAF	Nearest Gene	Consequence	Meta-analysis OR (95% CI)	*P*-value	FinnGen OR (95% CI)	UKBB OR (95% CI)	EstBB OR (95% CI)	*Q p*-value	*I*2
rs77249084	8	116560300	G	A	0.3	*EIF3H*	Regulatory region	1.31 (1.24–1.38)	1.78E–23	1.44 (1.33–1.56)	1.21 (1.09–1.34)	1.22 (1.1–1.34)	0.0074	0.8
rs11868207	17	68679579	C	T	0.23	*FAM20A (LINC01482)*	Intergenic (Intronic)	1.34 (1.26–1.42)	1.25E–22	1.46 (1.33–1.6)	1.29 (1.16–1.43)	1.24 (1.11–1.38)	0.056	0.65
rs12270054	11	65555077	T	C	0.22	*LTBP3*	Intronic	0.75 (0.71–0.8)	4.29E–20	0.68 (0.61–0.74)	0.76 (0.69–0.85)	0.86 (0.77–0.97)	0.0074	0.8
rs10592836	14	103471816	C	CAAT	0.34	*MARK3*	Intronic	1.25 (1.19−1.32)	2.79E–18	1.35 (1.25–1.46)	1.2 (1.09–1.32)	1.16 (1.06–1.28)	0.032	0.71
rs71900142	10	30775253	G	GCTTCTGGCTTAAGC	0.41	*ZNF438*	Intergenic	0.82 (0.78–0.86)	1.20E–15	0.75 (0.7–0.81)	0.88 (0.81–0.97)	0.86 (0.78–0.94)	0.015	0.76
rs7995158	13	110459370	G	A	0.55	*COL4A2 (COL4A2-AS2)*	3’ UTR (Intronic)	0.82 (0.79–0.86)	1.81E–15	0.83 (0.78–0.89)	0.79 (0.72–0.86)	0.85 (0.78–0.93)	0.47	0
rs39375	7	103837624	C	A	0.41	*RELN*	Intronic	1.21 (1.15–1.27)	3.21E–14	1.27 (1.18–1.37)	1.14 (1.04–1.25)	1.18 (1.08–1.29)	0.15	0.47
rs791903	6	33734868	C	G	0.5	*IP6K3*	Intronic	0.83 (0.8–0.88)	1.15E–13	0.84 (0.79–0.91)	0.82 (0.75–0.89)	0.84 (0.76–0.92)	0.85	0
*rs753138805*	*4*	*87845066*	*G*	*GGAAA*	*0.0033*	*MEPE*	*Frameshift*		*9.80E–14*	*21.5 (9.6–48.4)*	*NA*	*NA*	*NA*	*NA*
rs181831514	4	87901594	T	C	0.0024	*MEPE*	Intergenic	12.3 (6.09–24.9)	2.87E–12	19.66 (8.92–43.33)	NA	2.04 (0.43–9.62)	0.011	0.85
rs4917	3	186619924	C	T	0.64	*AHSG*	Missense	0.84 (0.8–0.88)	3.35E–12	0.86 (0.8–0.92)	0.77 (0.7–0.85)	0.88 (0.8–0.97)	0.12	0.52
rs13192457	6	44887654	A	C	0.44	*SUPT3H*	Intronic	1.21 (1.14–1.28)	4.80E–11	1.3 (1.21–1.39)	1.07 (0.98–1.18)	NA	0.0013	0.9
rs4636903	16	54995451	T	C	0.12	*IRX5*	Intergenic	1.28 (1.19–1.39)	7.32E–11	1.35 (1.2–1.52)	1.36 (1.18–1.56)	1.14 (0.99–1.31)	0.12	0.52
rs67284550	16	1480948	T	C	0.42	*PTX4*	Downstream gene	1.17 (1.12–1.23)	1.43E–10	1.25 (1.16–1.34)	1.13 (1.03–1.24)	1.09 (1–1.2)	0.055	0.66
rs11683921	2	111714056	G	T	0.38	*ANAPC1*	Intronic	1.17 (1.11–1.23)	3.23E–10	1.21 (1.12–1.3)	1.13 (1.03–1.24)	1.15 (1.05–1.27)	0.49	0
rs553652	11	96200151	A	G	0.92	*MAML2*	Intronic	1.3 (1.2–1.42)	9.24E–10	1.35 (1.2–1.52)	1.31 (1.08–1.6)	1.21 (1.04–1.42)	0.55	0
rs73172296	13	42525753	A	G	0.11	*TNFSF11*	Intergenic	1.26 (1.17–1.36)	1.18E–09	1.25 (1.13–1.4)	1.42 (1.2–1.68)	1.19 (1.03–1.36)	0.27	0.23
rs485107	8	8723898	G	C	0.53	*CLDN23*	Intergenic	1.16 (1.1–1.21)	2.20E–09	1.17 (1.09–1.26)	1.15 (1.05–1.26)	1.14 (1.04–1.25)	0.92	0
rs2762049	13	50248227	C	G	0.39	*KCNRG (DLEU1)*	Intergenic (Intronic)	1.16 (1.1–1.22)	2.34E–09	1.19 (1.11–1.28)	1.12 (1.02–1.23)	1.15 (1.05–1.26)	0.58	0
rs4877080	9	89398813	A	T	0.28	*SEMA4D*	Intronic	0.83 (0.78–0.88)	3.60E–09	0.84 (0.78–0.91)	0.81 (0.73–0.89)	NA	0.52	0
rs201694067	1	219593023	T	TGA	0.33	*ZC3H11B*	Intergenic	1.19 (1.12–1.26)	4.30E–09	1.18 (1.09–1.27)	NA	1.22 (1.11–1.34)	0.54	0
rs6066825	20	48723580	G	A	0.32	*PREX1*	Intronic	1.16 (1.1–1.22)	1.20E–08	1.17 (1.08–1.27)	1.25 (1.14–1.37)	1.06 (0.96–1.17)	0.066	0.63
rs66487118	19	10142669	A	G	0.3	*DNMT1*	Intronic	1.16 (1.1–1.23)	1.76E–08	1.14 (1.05–1.24)	1.26 (1.15–1.39)	1.1 (0.99–1.21)	0.12	0.53
rs8105161	19	41333726	C	T	0.16	*TGFB1*	Intronic	0.83 (0.78–0.89)	2.71E–08	0.81 (0.74–0.89)	0.85 (0.75–0.97)	0.86 (0.76–0.96)	0.71	0
rs2118612	15	67108152	T	C	0.79	*SMAD3*	Intronic	0.85 (0.8–0.9)	3.34E–08	0.84 (0.77–0.92)	0.81 (0.73–0.91)	0.89 (0.79–0.99)	0.59	0
rs4464751	6	73707844	T	G	0.54	*CD109*	Intronic	1.14 (1.09–1.2)	3.58E–08	1.15 (1.07–1.24)	1.18 (1.07–1.29)	1.1 (1.01–1.2)	0.58	0
rs80339979	9	99889430	G	GA	0.64	*STX17 (STX17-AS1)*	Intergenic (Intronic)	1.15 (1.09–1.21)	3.66E–08	1.1 (1.03–1.19)	1.16 (1.05–1.27)	1.22 (1.11–1.34)	0.26	0.27
rs6066131	20	46941020	C	T	0.45	*EYA2*	Intronic	1.14 (1.09–1.2)	4.91E–08	1.17 (1.09–1.26)	1.13 (1.03–1.24)	1.11 (1.01–1.21)	0.57	0

A total of 3,504 cases and 861,198 controls from FinnGen, Estonian Biobank and UK Biobank were included in the meta-analysis. A Bonferroni-corrected two-sided genome-wide p-value threshold of 5×10^−8^ was used to establish significance, accounting for multiple comparisons. For all loci, the nearest protein-coding gene is shown. RNA and antisense genes overlapping the lead variant are additionally shown in parentheses. In addition to the 27 lead variants from the meta-analysis, data (in italic) are presented for the frameshift variant rs753138805 in *MEPE* which was observed only in FinnGen and was not included in the meta-analysis. The *p*-value for Cochran’s *Q* test and the *I*^2^ statistic are reported for each lead variant. *Chr* chromosome, *EA* effect allele, *EAF* effect allele frequency, *NEA* non-effect allele, *OR* odds ratio, *CI* confidence interval.

Two of the GWAS meta-analysis lead variants (Near *MEPE* and *SUPT3H*) are driven by their association in FinnGen. The variant rs181831514 near *MEPE* is strongly enriched in Finland, with very low frequency in EstBB and UKBB. The lead variant rs13192457 in the association locus overlapping *SUPT3H* was not replicated at nominal significance in UKBB (*p* > 0.05) and was not included in EstBB (Supplementary Data 7). However, the second-most significant variant in the *SUPT3H* locus (rs12204678, 6:45153962:C > T, meta-analysis *p* = 8.3 × 10^−11^, MAF = 41%, OR = 0.85 [0.81–0.9]) was included in EstBB and was associated with otosclerosis at nominal significance (*p* = 0.029, OR = 0.90 [0.86-0.95]).

In two of the 27 genome-wide significant loci, the nearest genes (*MEPE* and *TGFB1*) have been implicated in candidate gene studies. One locus (denoted by the lead variant rs4464751 in an intron of *CD109*) is a previously identified linkage locus (OTSC7, 6q13–16.1)^25^. To our knowledge, the remaining 23 loci have not been characterized in association with otosclerosis^18–22^.

### Characterization of the potential susceptibility loci for otosclerosis

The lead variant in the chromosome 3 locus, rs4917 T > C, is a missense variant in the exon 6 of *AHSG* (EAF = 64%, OR = 0.84 [0.80–0.88], *p* = 3.4 × 10^−12^). The variant results in a methionine to threonine conversion and is predicted to be tolerated/benign by the SIFT and PolyPhen algorithms with a scaled CADD score of 9.9.

In the chromosome 4 locus near the *MEPE* gene, the rare frameshift variant rs753138805 (EAF 0.3% in controls and 1.3% in cases) was the lead variant in the GWAS in FinnGen with an odds ratio of 21.5 (95% CI 9.6-48.4) (*p* = 9.8 × 10^−14^). The variant is 2.4-fold enriched in Finnish compared with non-Finnish Europeans based on sequence data in the Genome Aggregation Database^26^. It was not included in the meta-analysis due to poor imputation quality in EstBB (Imputation information score 0.28) and non-inclusion in UKBB genotype data.

Among all 1,452 variants which reached genome-wide significance in the meta-analysis, thirteen missense variants were significantly associated with otosclerosis (Supplementary Data 9). One, rs140145986 in *RIN1* (EAF = 8.6%, OR = 1.3 [1.19-1.41], *p* = 4.1 × 10^−10^), was classified as deleterious and probably damaging by the SIFT and PolyPhen algorithms respectively, with a scaled CADD score of 23.1 (Supplementary Fig. 3).

Next, based on linkage disequilibrium in the FinnGen cohort, we examined 1142 variants (including those with AF < 0.001) in high LD (r^2^ > 0.6) with the lead variants from the meta-analysis. We observed one additional protein-altering variant, rs11526468 in *SEMA4D* (EAF = 30%, OR = 0.91 [0.87-0.96], *p* = 0.00039; Supplementary Data 10; Supplementary Fig. 3). The variant, resulting in an alanine to threonine conversion, is predicted to deleterious and probably damaging by the SIFT and PolyPhen algorithms, respectively, with a scaled CADD score of 24.3.

Finally, the lead variants were intronic for protein-coding genes in fourteen loci (Table 2). The functional assessment of such variation is more challenging. In the locus on chromosome 7, the strongest association was observed for variants in the second and third introns of *RELN* concordant with previous GWAS^17,18^. In a locus on chromosome 19, the association signal overlapped with several genes including *TGFB1*, *AXL*, *HNRNPUL1* and *CCDC97*, with the only genome-wide significant association observed for the intronic *TGFB1* variant rs8105161 (EAF = 16%, OR = 0.83 [0.78-0.89], *p* = 2.7 × 10^−8^). On chromosome 6, the association signal only overlapped the *CD109* gene within the wider OTSC7 linkage region. In two of the significant loci, the associated variants were tightly clustered in the region of antisense genes: *COL4A2-AS2* and an intronic region of *COL4A2* on chromosome 13, and *STX-17-AS1* on chromosome 9. On chromosome 6, an apparent association haplotype block spanned the entire *SUPT3H* gene and the first exon and first intron of the *RUNX2* gene; the lead variant rs13192457 (EAF = 44%, OR = 1.21 [1.14–1.28], *p* = 4.8 × 10^−11^) is located in an intronic region of *SUPT3H*.

### Fine-mapping of the association loci

To identify the most likely causal variants in the otosclerosis association loci, we performed fine-mapping analyses in FinnGen using the SuSie software v.1.0^27^. We analyzed all loci that had suggestive evidence of association in FinnGen (*p* < 1 × 10^−6^). The number of variants in good-quality credible sets ranged from three (for the chromosome 4 locus near *MEPE*) to 156. (Supplementary Data 11 and 12). In the chromosome 4 locus, the *MEPE* frameshift variant rs753138805 was the most likely causal variant (posterior inclusion probability [PIP] = 40%) (Supplementary Data 12). No other coding variants identified in previous analyses (e.g. rs140145986 in *RIN1* or rs11526468 in *SEMA4D*) were highlighted by fine-mapping. In the chromosome 16 locus near *PTX4*, all credible set variants were located upstream of or within the first intron of *CLCN7*, a gene associated with osteopetrosis, but the significance of this finding is uncertain^28^.

### Phenome-wide association studies of the 27 otosclerosis lead variants

To assess pleiotropic effects of genetic risk factors to otosclerosis, we examined the phenome-wide associations of the 27 lead variants from the meta-analysis with a total of 4756 traits registered in the GWAS Atlas. Lead variants were frequently associated with several traits at the bonferroni-corrected threshold of *p* < 1.05×10^−5^: twelve lead variants were associated with heel bone mineral density (BMD), six with sitting height, four with standing height, three with hip osteoarthritis, three with FEV1/FVC ratio, three with trunk impedance measures (fat-free mass, trunk predicted mass and impedance of arm), and three with body mass index (Supplementary Data 13 and 14). We observed significant discordance between the direction of effect for otosclerosis and heel bone mineral density when evaluating all variants reaching nominal significance in GWAS Atlas (2 directionally concordant co-associations and 15 directionally discordant co-associations, *p*-value = 0.0023; Supplementary Data 15).

As we found no reported associations in the GWAS Atlas for the rare *MEPE* rs753138805 frameshift variant, we performed a separate phenome-wide association study for the variant in FinnGen. Among 2,861 total traits, in addition to otosclerosis and hearing loss, rs753138805 was significantly associated with fractures of the lower leg (beta = 0.53, *p* = 1.5 × 10^−5^, Supplementary Data 16).

We also examined the correlation of effect estimates for otosclerosis and recurring traits from the PheWAS for all meta-analysis lead variants in the FinnGen cohort. Bone mineral density was not available, but we could evaluate effect estimates for osteoporosis and risk of fractures. None of the associations remained statistically significant after correction for multiple comparison (Supplementary Fig. 4).

### Analyses of shared heritability

Following frequently observed phenotypes in the phenome-wide association analysis of the lead variants, we tested the genetic correlations of otosclerosis with 54 bone-related, anthropometric and lung traits in LDHub^23,24,29^. None of the traits were significantly genetically correlated with otosclerosis after multiple correction (*p* > 0.00093 for all traits) (Supplementary Data 17).

### Rare disease associations in GWAS Catalog and ClinVar

We queried the GWAS Catalog and ClinVar database for rare disease associations for all 1452 variants associated with otosclerosis at genome-wide significance (*p* < 5 × 10^−8^). In the chromosome 8 locus overlapping *EIF3H*, rs13279799 (OR for otosclerosis = 0.77 [0.71–0.83]; *p* = 2.8×10^−11^) has previously been associated with ossification of the longitudinal ligament of spine, a rare form of heterotopic ossification (Supplementary Data 18)^30^. Both rs13279799 and the fine-mapped credible set were located downstream of *EIF3H*. None of the 1452 variants which were associated with otosclerosis at genome-wide significance, nor any variants associated with otosclerosis at nominal significance (*p* < 0.05) within the association loci, were reported as pathogenic for diseases registered in ClinVar.

### Gene-based and gene set analysis

In a gene-based analysis using MAGMA, a total of 53 genes were significantly associated with otosclerosis (*p* < 2.65 × 10^−6^) (Supplementary Data 19). The gene-based associations were significantly enriched (*p* < 4.9 × 10^−6^) for two gene sets based on gene ontology terms: COLLAGEN TYPE IV TRIMER (*p* = 1.9 × 10^−6^) and BASEMENT MEMBRANE COLLAGEN TRIMER (*p* = 1.2 × 10^−6^).

In a GO Biological Process (BP) enrichment analysis of the 27 protein-coding genes nearest to the lead variants, we found enrichment of nine main BP categories and a total of 16 BPs (false discovery rate corrected *p*-value < 0.05) (Supplementary Data 20). All enriched categories were relevant for bone metabolism, biomineralization, skeletal system development, positive regulation of cell development, or TGFβ receptor signalling.

### Replication of previous otosclerosis candidate genes and linkage loci

As two of the genome-wide significant loci from the meta-analysis overlapped with candidate genes (*TGFB1* and *MEPE*) and one association locus overlapped with the OTSC7 linkage locus, we also examined the association signals near other candidate genes and linkage loci (Supplementary Data 21; Supplementary Fig. 5). With the exception of the *RELN* variant rs3914132 identified in the previous GWAS study, we observed no significant associations for previously reported susceptibility variants including the recently reported *FOXL1* deletion (AF = 0.002, OR = 1.11 [0.62-2], *p* = 0.72)^16,31–33^. None of the previously reported susceptibility variants in *SERPINF1* or *ACAN* were included in the current meta-analysis, but we observed no suggestive association signals (*p* < 1 × 10^−6^) in a 3MB window surrounding *SERPINF1* or *ACAN*^12,13^. Finally, we assessed all previously reported linkage loci, but found no suggestive associations with the exception of the genome-wide significant association within OTSC7.

### Colocalization

As the risk loci could alter otosclerosis susceptibility via gene expression, we evaluated colocalization between the otosclerosis meta-analysis and expression datasets available in the eQTL catalogue. As no data on bone expression was available, we focused on genes with altered expression across at least two tissue types with a causal posterior probability of ≥ 0.1%. Fourteen such genes were identified (Supplementary Data 22 and 23). Otosclerosis colocalized genetically with the expression of *BANF1*, *MARK3* and *SUPT3H* in the highest number of tissues (14, 8 and 6, respectively), while the highest causal posterior probability was observed for *TELO2* (9.5%), *ZNRD2* (6.3%), *EHBP1L1* (6.0%).

### Validation of otosclerosis gene candidates in the mammalian cochlea

**Fig. 2:**
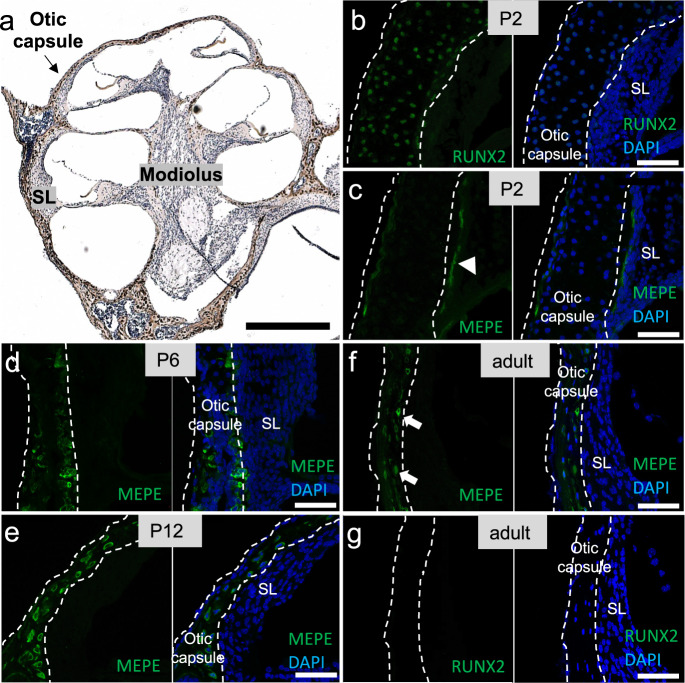
Localization of RUNX2 and MEPE proteins in the murine cochlea. **a** Cross-section through a P12 mouse cochlea stained with anti-MEPE-antibody (brown) and counterstained with hematoxylin (blue). **b** Immunofluorescent staining for RUNX2 (green) was observed in the nuclei (blue) of P2 osteoblasts. **g** Lack of RUNX2 expression in the adult otic capsule. **c** – **f** Immunofluorescence for MEPE (green) developed from more diffuse (**c** white arrowhead) to more clearly cellularly localized (**f** white arrows) with age. SL Spiral ligament. Representative images of *N* = 3 animals per age group. Scale bars: a: 500 µm, b–g 50 µm.

To investigate the pattern of expression of candidate genes, we performed immunostaining on neonatal and adult mouse cochleae for the proteins encoded by *RUNX2* and *MEPE* genes. We found strong nuclear expression of RUNX2 in osteoblasts within the otic capsule only on postnatal day 2 (P2)-old mice (Fig. 2b) and the absence of RUNX2 immunostaining in the adult inner ear (Fig. 2g). Immunostaining for MEPE revealed dynamic changes through postnatal development: the pattern was more diffuse at P2 (Fig. 2c), then limited to maturing osteocytes within the otic capsule of postnatal day 6 (P6)-old mice (Fig. 2d), and persisted in mature osteocytes of postnatal day 12 (P12)-old mice (Fig. 2a, e) and 3-month-old mice (Fig. 2f).

## Discussion

Although otosclerosis is highly heritable its genetic background is still poorly understood. Previous studies have reported one GWAS locus (*RELN*), while results for candidate genes have been inconclusive. Here, we identify 27 loci associated with otosclerosis, of which 23 are novel. We also identify four previously reported loci: the *RELN* locus, the loci of two candidate genes (*TGFB1* and *MEPE)* and the OTSC7 linkage locus. These results offer further insights to disease pathophysiology, especially when interpreted in light of the dynamic communication between the bony otic capsule and the intracochlear soft tissues encased by this bone. Specifically, unlike most other bones in the body that continuously remodel throughout life, the normal adult otic capsule remodels minimally if at all^34^. This lack of remodeling is achieved, at least in part, through molecules secreted by intracochlear soft tissues, such as osteoprotegerin (OPG), which diffuse into the surrounding otic capsule^35,36^.

The novel loci we identified are highly relevant because of known histopathologic and clinical features of otosclerosis. Histologically, otosclerosis is characterized by pathologic bone remodelling that starts at sites of predilection, which are globuli interossei, embryonic rests of cells from the original cartilaginous framework since the otic capsule is formed through endochondral ossification. Clinically, otosclerosis is similar to other diseases that cause stapes fixation, such as osteogenesis imperfecta, multiple-synostoses syndrome and congenital X-linked deafness with stapes gusher; the genes identified in our study are related to the genes known to cause stapes fixation in other diseases.

We confirm a polygenic basis to otosclerosis. Early genetic studies of otosclerosis assumed a dominant inheritance, but modest success in mapping otosclerosis to one or a few high-impact genes, even in family based linkage studies, has suggested a complex nature. At an individual level, genetic disease susceptibility could reflect the contribution of multiple low-impact or few high-impact variants. Many etiologies to otosclerosis have been proposed, including differences in TGFβ, parathyroid hormone or angiotensin II signalling, alterations in collagen type I, inflammation, viral infection, and autoimmunity^37^. The association loci include several genes involved in the regulation of bone structure; we validated two of these genes, *RUNX2* and *MEPE*, via immunostaining of inner ear sections for the corresponding proteins. Follow-up functional studies are expected to reveal additional molecular insights.

In a considerable number of the loci, either the nearest protein-coding gene or a gene whose expression colocalizes genetically with otosclerosis is associated with severe skeletal disorders (Supplementary Table 1). Disordered or heterotopic bone growth is a common feature in diaphyseal dysplasia (as caused by mutations in *TGFB1*), osteopetrosis subtypes (as caused by mutations in *CLCN7* and *TNFSF11* which codes for RANKL) and ossification of the posterior longitudinal ligament of spine (previously associated with rs13279799 for which we report an association with otosclerosis). The cochlear RANKL-RANK-OPG axis is critical for regulation of not only bone remodeling within the otic capsule, but also neuronal survival and outgrowth^35,36,38,39^. Compared with mono- or oligogenic severe skeletal dysplasias, otosclerosis presents later in adulthood and represents a more limited and common phenotype. Genetic susceptibility to otosclerosis in the population may reflect more moderate and aggregate polygenic disruption of bone growth and regulation.

The effects on bone growth and remodeling in otosclerosis may be mediated by several pathways. The known biological functions of many genes within the otosclerosis association loci include regulation of osteoblasts – the cells responsible for bone formation – and osteoclasts – responsible for the breakdown of bone, either through effects on cell differentiation or function (Supplementary Table 1). Some genes (e.g. *AHSG*, *IRX5*, *MEPE*, *FAM20A* and *SMAD3*) are also directly involved in biomineralization, and *AHSG* and the candidate gene *MEPE* have been reported to have dual roles in the regulation of mineralization and osteoclast, osteoblast or osteocyte cell lineages. Our localization of MEPE protein within the maturing and adult otic capsule validates our GWAS finding.

Recently, Schrauwen and colleagues proposed a model of otosclerosis in which increased bone turnover results from mutations ablating two functional motifs of *MEPE*, leading to both accelerated osteoclast differentiation and enhanced mineralization and thus increased bone turnover^14^. In contrast with otosclerosis, they observed frameshift mutations ablating only one functional motif of *MEPE* in individuals with a severe craniofacial defect with thickening of the skull, potentially reflecting an unopposed increase in mineralization. Here, we report a genome-wide significant association in FinnGen between otosclerosis and the frameshift mutation rs753138805 that is likely to ablate both functional motifs in *MEPE*. The same mutation was identified in two families (Dutch and Belgian) and nine unrelated cases by Schrauwen and colleagues^14^. Reflecting a systemic effect on the skeleton, the rs753138805 variant is also associated with increased risk of leg fractures^40,41^. The identification of such a Finnish-enriched high-impact variant is likely possible due to a relatively recent population bottleneck event and follows similar reports for other traits^42–44^. An inherent shortcoming is that replicating variants that are rare in other populations is challenging in a GWAS setting. Yet, previous literature combined with the genetic association and immunostaining findings in this study elevate *MEPE* as a likely high-impact susceptibility gene for otosclerosis.

Several genes in the susceptibility loci converge on transforming growth factor beta receptor signalling pathways. *TGFB1* has long been proposed as a susceptibility gene for otosclerosis, but the quality of the supporting evidence has been relatively weak^17–21,32^. Here, we report an association at the *TGFB1* locus at genome-wide significance, with the strongest association for the intronic variant rs8105161.

TGFβ1, a member of the transforming growth factor beta superfamily, is a cytokine with an essential role in skeletal development and mature bone remodeling, regulating both osteoblast and osteoclast cell lineages^45,46^. Gain-of-function mutations in *TGFB1* predispose to diaphyseal dysplasia, and mutations in other TGFβ superfamily members can cause multiple skeletal disorders of varying severity^47^. In the mouse inner ear, TGFβ1 is abundantly expressed in stria vascularis and supporting cells of the sensorineural epithelium^48^. As a secreted molecule, TGFβ1 could diffuse from these cells into the surrounding otic capsule to regulate its remodeling in a paracrine fashion. In support of this hypothesis, TGFβ inducer, called TGFBI is abundant in human inner ear fluid, called perilymph, while mRNA encoding TGFBI is localized in murine cochlear neurons and hair cells^49^. TGFβ1 is also produced locally within the murine otic capsule where *Tgfb1* mRNA levels are significantly higher than in the parietal bone^36^.

Other genes relevant for TGFβ signalling include *SMAD3*, *CD109*, *LTBP3*, and *AHSG*, all of which are nearest to the lead variants in their respective loci, and *RUNX2* which partly overlaps an association signal (Supplementary Table 1). Smad3 is a downstream transcription factor in the TGFβ signalling pathway; mutations in *SMAD3* can cause an aneurysms-osteoarthritis syndrome with features of craniofacial and skeletal abnormalities^50^. RUNX2 is a transcription factor regulated by TGFβ1-signalling via both the canonical Smad and p38 MAPK pathways and has an essential role in osteoblast and chondrocyte differentiation^45,46,51^. Relevantly, we found RUNX2 expression in the developing but not adult otic capsule (Fig. 2). This finding is significant because otosclerosis is associated with embryonic cell rests within the otic capsule, which are unique to the human temporal bone. CD109 is a TGF-β co-receptor and negative regulator of TGF-β signalling^52^. Latent-transforming growth factor beta-binding protein 3 (LTBP3) can regulate the latency and activation of TGFβ1 through direct extracellular binding^53^. Alpha2-HS-glycoprotein/fetuin (AHSG) antagonizes TGFβ1-signalling by binding to TGFβ1 and TGFβ related bone morphogenic proteins (BMPs)^54,55^. AHSG can also affect mineralization directly by inhibiting calcium phosphate precipitation via formation of calcium-fetuin complexes^56–58^. Importantly, mutations in noggin, which is an inhibitor of several BMPs, are associated with multiple-synostoses syndrome whose otologic presentation mimics otosclerosis and is characterized by stapes fixation^59^.

In addition to signaling proteins, *COL1A1* coding for the major subunit of type I collagen is a prominent candidate gene for otosclerosis^11,31–33,60^. Mutations in *COL1A1* predispose to osteogenesis imperfecta, often also characterized by hearing loss. In osteogenesis imperfecta, similarly to carriers of truncating *MEPE* variants, increased bone turnover in the middle ear could occur in association with general skeletal fragility. While we do not observe a strong signal for *COL1A1*, similarly to most examined candidate genes, we report an intronic association signal in *COL4A2*, coding for a subunit of collagen IV, located in the basement membrane. Future mechanistic studies could examine whether *COL4A2* has a structural or signaling role in otosclerosis. *COL4A2* is highly conserved across species^61^. We are not aware of a previous association with skeletal disorders. However, mutations in *COL4A3-5* genes are known to cause Alport’s syndrome, which manifests as progressive sensorineural hearing loss and nephritis. Histopathologically, Alport’s syndrome is characterized by abnormalities of the cochlear basement membrane supporting the organ of Corti and dysmorphogenesis of the organ of Corti^62^. These alterations are thought to affect cochlear micromechanics. While the distribution of type IV collagen alpha 1, alpha 3 and alpha 5 chains has been described in the human cochlea, the distribution of alpha 2 chain remains to be determined^63^. Although type IV collagens regulate BMPs in Drosophila, a similar role in humans is uncertain^64^.

Our results highlight several genes and signaling pathways for follow-up mechanistic studies. Genetic discovery has also increasingly preceded or guided therapeutic development. Future sequencing studies could aim to discover loss-of-function mutations within the susceptibility loci to approximate the effects of therapeutic inhibition. In the case of *TGFB1*, loss-of-function mutations are exceedingly rare and functional studies are likely needed to assess the effect of direct TGFβ1 inhibition^26^. Of note, an inhibitor of BMP type I receptor kinases has been studied for the treatment of ectopic ossification; however, in our meta-analysis, its targets (coded by *ACVR1* and *BMPR1A)* were not significantly associated with otosclerosis^65^. Reflecting the heterogeneity of conditions associated with different TGFβ superfamily genes, therapeutic intervention may need to be precisely tailored to each condition^45^.

Although we present the largest GWAS study of otosclerosis to date, our study has limitations. The identification of otosclerosis cases is based on ICD diagnoses, which we could not verify with audiometric or imaging data. The prevalence of otosclerosis was slightly higher in FinnGen and EstBB than the reported prevalence in Western populations. This may be explained by the ascertainment of cases from hospital and specialist outpatient settings for these biobanks. The prevalence of stapes procedures among cases in FinnGen and EstBB is concordant with clinical experience. Case ascertainment in UKBB was more reliant on self-reported and procedure-based data compared with FinnGen and EstBB. Statistical colocalization analyses relied on expression data from non-bone tissues; associations in bone could differ. Although nearest genes to lead variants are causal more often than expected by chance, the physical proximity of lead variants with biologically relevant genes does not prove causation; identified and unidentified variants in the loci can exert their effects through yet unknown mechanisms^67^.

In summary, we determine a polygenic basis to otosclerosis with 27 genome-wide significant susceptibility loci. Analysis of the association loci suggests potential avenues for understanding disease pathophysiology through genes involved in bone remodeling, mineralization, and basement membrane collagen composition. In particular, our results highlight several genes involved in TGFβ signalling for follow-up studies.

## Methods

### Cohort descriptions

We identified individuals with ICD-based otosclerosis diagnoses from three national biobank-based cohorts: The Finnish FinnGen cohort, the Estonian Biobank (EstBB) and the UK Biobank (UKBB). Written informed consent was obtained from all study participants at recruitment.

The FinnGen data used here comprise 250,844 individuals from FinnGen Data Freeze 6 (https://www.finngen.fi/en)^66^. The data were linked by unique national personal identification numbers to the national hospital discharge registry (available from 1968) and the specialist outpatient registry (available from 1998). Data comprised in FinnGen Data Freeze 6 are administered by regional biobanks (Auria Biobank, Biobank of Central Finland, Biobank of Eastern Finland, Borealis Biobank, Helsinki Biobank, Tampere Biobank), the Blood Service Biobank, the Terveystalo Biobank, and biobanks administered by the Finnish Institute for Health and Welfare (THL) for the following studies: Botnia, Corogene, FinHealth 2017, FinIPF, FINRISK 1992–2012, GeneRisk, Health 2000, Health 2011, Kuusamo, Migraine, Super, T1D, and Twins). Patients and control subjects in FinnGen provided informed consent for biobank research, based on the Finnish Biobank Act. Alternatively, older research cohorts, collected prior the start of FinnGen (in August 2017), were collected based on study-specific consents and later transferred to the Finnish biobanks after approval by Fimea, the National Supervisory Authority for Welfare and Health. Recruitment protocols followed the biobank protocols approved by Fimea. The Coordinating Ethics Committee of the Hospital District of Helsinki and Uusimaa (HUS) approved the FinnGen study protocol Nr HUS/990/2017.

The FinnGen study is approved by Finnish Institute for Health and Welfare (permit numbers: THL/2031/6.02.00/2017, THL/1101/5.05.00/2017, THL/341/6.02.00/2018, THL/2222/6.02.00/2018, THL/283/6.02.00/2019, THL/1721/5.05.00/2019, THL/1524/5.05.00/2020, and THL/2364/14.02/2020), Digital and population data service agency (permit numbers: VRK43431/2017-3, VRK/6909/2018-3, VRK/4415/2019-3), the Social Insurance Institution (permit numbers: KELA 58/522/2017, KELA 131/522/2018, KELA 70/522/2019, KELA 98/522/2019, KELA 138/522/2019, KELA 2/522/2020, KELA 16/522/2020 and Statistics Finland (permit numbers: TK-53-1041-17 and TK-53-90-20).

The Biobank Access Decisions for FinnGen samples and data utilized in FinnGen Data Freeze 6 include: THL Biobank BB2017_55, BB2017_111, BB2018_19, BB_2018_34, BB_2018_67, BB2018_71, BB2019_7, BB2019_8, BB2019_26, BB2020_1, Finnish Red Cross Blood Service Biobank 7.12.2017, Helsinki Biobank HUS/359/2017, Auria Biobank AB17-5154, Biobank Borealis of Northern Finland_2017_1013, Biobank of Eastern Finland 1186/2018, Finnish Clinical Biobank Tampere MH0004, Central Finland Biobank 1-2017, and Terveystalo Biobank STB 2018001.

EstBB is a population-based cohort of 200,000 participants with a rich variety of phenotypic and health-related information collected for each individual^68^. At recruitment, participants have signed a consent to allow follow-up linkage of their electronic health records (EHR), thereby providing a longitudinal collection of phenotypic information. EstBB allows access to the records of the national Health Insurance Fund Treatment Bills (from 2004), Tartu University Hospital (from 2008), and North Estonia Medical Center (from 2005). For every participant there is information on diagnoses in ICD-10 coding and drug dispensing data, including drug ATC codes, prescription status and purchase date (if available). The study has obtained approval from the Ethics Review Committee on Human Research of the University of Tartu.

UKBB comprises phenotype data from 500,000 volunteer participants from the UK population aged between 40 and 69 years during recruitment in 2006–2010^69^. Data for all participants have been linked with national Hospital Episode Statistics. UK Biobank has approval from the North West Multi-centre Research Ethics Committee (MREC) as a Research Tissue Bank (RTB) approval. The analyses for this study have been conducted under UK Biobank Application Numbers 31063 and 22627.

### Identification of otosclerosis cases

Case status was assigned for individuals with any ICD-10 H80* code, the ICD-9 code 387, or the ICD-8 code 386 (Table 1), jointly corresponding to the Phecode 383. In FinnGen, only ICD codes registered in the specialty care setting were used for case definition. In UKBB, due to a large number of likely cases without ICD codes, cases were additionally ascertained based on self-reported otosclerosis diagnoses (code 20002), self-reported stapedectomy operation codes (code 20004), and the OPCS4 codes D171 (stapedectomy) and D172 (revision of stapedectomy). No self-reported data was available in FinnGen or EstBB. In FinnGen, stapes procedures were identified based on the Nomesco codes DDA00 (stapedotomy) and DDB00 (stapedectomy), and in EstBB, based on the national health insurance treatment service code 61006 (stapedotomy). Individuals not meeting these otosclerosis case definitions were designated as controls.

### Genotyping and imputation of variants

FinnGen samples were genotyped using Illumina and Affymetrix arrays (Illumina Inc., San Diego, and Thermo Fisher Scientific, Santa Clara, CA, USA). Genotype imputation was performed using a population-specific SISu v3 imputation reference panel comprised of 3,775 whole genomes as described in a public protocol (https://www.protocols.io/view/genotype-imputation-workflow-v3-0-xbgfijw). We validated the imputation of the rs753138805 variant in *MEPE* within the Migraine Family subcohort of FinnGen. The primers were designed with the primer3 software v1.1.4. The left (forward) primer sequence was TCCATGAAACCTGATTTGACCA and right (reverse) primer sequence was CCCAGGAGTTTAATCGCAGT. Among 65 individuals determined as heterozygous for rs753138805 by imputation, we confirmed the genotype by Sanger sequencing in 56 individuals (86%). Five carriers predicted to be homozygous for the reference allele were examined as negative controls and their genotypes were confirmed by Sanger sequencing.

The samples from the Estonian Biobank were genotyped at the Genotyping Core Facility of the Institute of Genomics, University of Tartu using the Global Screening Array (GSAv1.0, GSAv2.0, and GSAv2.0_EST) from Illumina. Altogether 206,448 samples were genotyped and PLINK format files were exported using GenomeStudio v2.0.4^70^. Individuals were excluded from the analysis if their call-rate was < 95% or if the sex defined based on heterozygosity of the X chromosome did not match the sex in phenotype data. Variants were excluded if the call-rate was < 95% or HWE p-value was < 1e-4 (autosomal variants only). Variant positions were updated to genome build 37 and all alleles were switched to the TOP strand using tools and reference files provided at https://www.well.ox.ac.uk/~wrayner/strand/. After QC the dataset contained 202,910 samples for imputation. Before imputation variants with MAF < 1% and indels were removed. Prephasing was done using the Eagle v2.3 software^71^. The number of conditioning haplotypes Eagle2 uses when phasing each sample was set to: --Kpbwt=20000. Imputation was done using Beagle v.28Sep18.793 with effective population size ne=20,000^72^. An Estonian population specific imputation reference of 2297 WGS samples was used^73^.

UKBB samples have been genotyped and imputed and released for research use previously^69^. In brief, genotyping of stored blood samples was carried out by Affymetrix Research Services Laboratory, and Affymetrix applied a custom genotype calling pipeline and quality filtering. Approximately 850,000 variants were directly genotyped. After imputation of over 90 million variants, a total of 97,059,328 UKBB version 3 imputed variants were released and were included in this study prior to further quality controls detailed below.

### Statistical analysis

GWAS for the individual study cohorts was performed using a generalized mixed model with the saddlepoint approximation using SAIGE v0.20, using a kinship matrix as a random effect and covariates as fixed effects^74^. For all cohorts, variants with a minor allele frequency less than 0.1% were excluded.

In FinnGen, samples from individuals with non-Finnish ancestry and twin/duplicate samples were excluded, and GWAS was performed for 1,563 cases and 249,281 controls. Age, sex, 10 PCs and the genotyping batch (for batches with at least 10 cases and controls) were used as covariates. Results were filtered to variants with imputation INFO score > 0.6. In addition to the primary GWAS, we performed additional sensitivity analyses in FinnGen to evaluate the effect of gender and control selection (Supplementary Fig. 6 and Supplementary Data 24-26). Separate GWAS were conducted for women (1002 cases and 140,222 controls) and men (561 cases and 109,059 controls) to evaluate potential differences in effect directions, which were not observed. To evaluate the potential effect of undiagnosed cases among controls, GWAS were also performed comparing all 1,563 cases with controls filtered to include only individuals over age 65 (110,166 controls), individuals without any ICD-based diagnosis of hearing loss (231,502 controls) and individuals over age 65 without any ICD-based diagnosis of hearing loss (96,564 controls). Due to the similarity of the GWAS signals for different control definitions, we continued with the initial control definition for all subsequent analyses in each cohort.

In EstBB, a GWAS was performed on 985 cases and 196,516 controls of European ancestry including related individuals and adjusting for the first 10 PCs of the genotype matrix, as well as for birth year and sex.

In UKBB, European ancestry classification and genomic analyses were performed identically to the approach in the Pan UKBB study (https://pan.ukbb.broadinstitute.org/docs/qc). Case and control selection were refined specifically for this study. Samples of European ancestry were identified via a two-stage approach. First, continental ancestry was assigned based on principal component (PC) analysis on unrelated individuals from a combined 1000 Genomes and Human Genome Diversity Panel reference dataset. PC loadings were used to project UKBB individuals into the same PC space. A random forest classifier was trained based on continental ancestry meta-data and top 6 PCs from the reference training data. Second, assigned ancestry classifications were further refined by pruning outliers within each continental assignment by plotting histograms of individual distances from population centroids calculated across 10 PCs. From all version 3 imputed variants, a total of 29,865,259 with INFO score > 0.8 and an allele count of at least 20 in each population were retained for the GWAS with SAIGE. Age, sex, age*sex, age^2^, age^2^*sex and the first 10 PCs were used as GWAS covariates.

Approximate estimates for the narrow sense heritability of otosclerosis in each cohort and the subsequent meta-analysis were obtained and compared using three different summary statistics based methods: 1) LD Score Regression (LDSC) v1.0.1, and 2) BLK-LDAK and 3) LDAK-Thin of the SumHer software in LDAK v5.0^23,24,75^. We used summary statistics separately from the meta-analysis and each cohort, restricting the analyses to variants present in HapMap3 (https://www.sanger.ac.uk/resources/downloads/human/hapmap3.html)^76^. For the BLK-LDAK and LDAK-Thin models, we used the European tagging files based on UKBB (http://dougspeed.com/pre-computed-tagging-files/), and for LDSC we used LD Scores computed using 1000 Genomes European data (https://data.broadinstitute.org/alkesgroup/LDSCORE/eur_w_ld_chr.tar.bz2). For the liability transformations, a population prevalence approximation of 0.3% was used.

Variant positions in the UKBB and EstBB summary statistics were lifted from GRCh37 to GRCh38 using the liftOver v1.12 Bioconductor R package^77^. To assess similarity of genetic effects between cohorts, we used the cross-trait LD Score Regression (LDSC) v1.0.1 to calculate pairwise genetic correlations (r_*g*_) based on summary statistics from each cohort for variants present in HapMap3^23,24^.

Summary statistics from the individual cohorts for 13,615,309 variants present in at least two cohorts with a cross-cohort minor allele frequency > 0.1% and imputation INFO score ≥ 0.7 were combined using an inverse-variance weighted fixed-effect meta-analysis with GWAMA v2.2.2^78^. The following analyses are based on summary statistics from the meta-analysis unless otherwise specified.

### Characterization of association loci

For the individual cohorts and meta-analysis, we merged genome-wide significant variants within 1.5 Mb of each other into association loci. The lead variants from these loci were not in LD with each other (r^2^ < 1 × 10^−4^ for all variant pair comparisons in FinnGen). We annotated the lead variants by mapping their physical position to genes and consequences using the Ensembl Variant Effect Predictor (VEP) based on the GRCh38 genome build (https://useast.ensembl.org/info/docs/tools/vep/index.html)^79^. In addition, we annotated all genome-wide significant variants and all variants in high LD (r^2^ > 0.6) with the lead variants from the meta-analysis using VEP. As the highest number of association loci was observed in FinnGen, we estimated LD in the FinnGen cohort using PLINK v1.07^80,81^. LocusZoom was used to visualize the association loci and the regions surrounding other genes of interest^82^.

### Fine-mapping

Based on otosclerosis summary statistics and linkage disequilibirium information from FinnGen, we fine-mapped all regions where the lead variant reached a p-value of < 1 × 10^−6^ in FinnGen using SuSiE^27^. In-sample dosage LD was computed using LDStore2 for each fine-mapping region. We used a 3 Mb window (±1.5 Mb) around each lead variant (merging overlapping regions) with 10 as the maximum number of causal variants in a locus.

### Phenome-wide association analyses and genetic correlations

For all 27 lead variants from genome-wide significant association loci in the meta-analysis, we queried the GWAS Atlas database (https://atlas.ctglab.nl) for significant associations among 4756 reported traits^83^. GWAS Atlas is a comprehensive database of publicly available GWAS summary statistics. We catalogued traits which were significantly associated with the lead variants at the Bonferroni-adjusted threshold of *p* < 1.05 × 10^−5^.

We also evaluated potential directional concordance or discordance of genetic effect estimates for recurring traits and otosclerosis using binomial tests with the null hypothesis that directional concordance and discordance are equally likely. All previously reported associations reaching nominal significance (*p*-value < 0.05) in GWAS Atlas were included, and seven traits which were associated with at least ten otosclerosis GWAS lead variants were identified (Supplementary Data 15). A Bonferroni-corrected *p*-value threshold of 0.05/7 was used to evaluate statistical significance in the binomial tests.

For the *MEPE* frameshift variant observable only in FinnGen and not included in GWAS Atlas, we performed a phenome-wide association analysis of 2861 registry-based disease endpoints in FinnGen^66^. A Bonferroni-corrected threshold α = 0.05/2861) was used to establish significance. GWAS for the endpoints was performed similarly to the GWAS for otosclerosis. The study participants were linked with national registries covering the whole population for hospital discharges (data available since 1968), deaths (1969–), outpatient specialist appointments (1998–), cancers (1953–), and medication reimbursements (1995–). Disease endpoints were collated based on International Classification of Diseases (ICD) codes (revisions 8–10), International Classification of Diseases for Oncology (ICD-O) third edition codes, NOMESCO procedure codes, Finnish-specific Social Insurance Institute (KELA) drug reimbursement codes, and drug-specific ATC-codes. In addition, specific clinical endpoints combining relevant comorbidities and exclusion criteria have been curated in coordination with clinical expert groups. Currently available FinnGen phenotype definitions are available online at https://www.finngen.fi/en/researchers/clinical-endpoints.

Following frequently observed associations of the meta-analysis lead variants in the phenome-wide association analysis, we tested the genetic correlations of otosclerosis with all available bone, anthropometric and lung traits in LDHub, a centralized database of summary-level GWAS results. Genetic correlation analyses were conducted using the automated pipeline after filtering the meta-analysis summary statistics to the HapMap3 variants recommended by the authors (v1.9.3.; http://ldsc.broadinstitute.org/ldhub/)^23,24,29^. A Bonferroni-corrected *p*-value threshold (α = 0.05/54) was used to evaluate statistical significance.

Finally, we queried the GWAS catalog (version 1.0.2) and the ClinVar database (https://www.ncbi.nlm.nih.gov/clinvar; accessed on 14 April 2021) for previous associations with rare diseases for a) any of the 1452 variants associated with otosclerosis at genome-wide significance, and b) any variants in the association loci which were associated with otosclerosis at nominal significance (*p* < 0.05).

### Gene and gene set -based analyses

We used MAGMA v1.08 to identify genes and gene sets associated with otosclerosis based on effect estimates from the meta-analysis^84^. Variants were mapped to 18,877 genes based on their RefSNP numbers. We performed a gene-based analysis using the default SNPwise-mean model and a Bonferroni-corrected *p*-value threshold (α = 0.05/18,877). Based on the results from the gene-based analysis, we then performed a competitive gene set based analysis using 10,182 GO term based gene sets downloaded from the Molecular Signature Database v7.1 (http://www.gsea-msigdb.org/gsea/msigdb), using a Bonferroni-corrected *p*-value threshold (α = 0.05/10,182)^85^. Set-specific QQ plots were produced by permutation analysis to evaluate the influence of outliers (Supplementary Fig. 7).

We performed a separate GO Biological Process (BP) enrichment analysis for the 27 protein-coding genes nearest to the lead variants from the meta-analysis. The analysis was performed using the online tool provided by the Gene Ontology Consortium connected to the PANTHER classification system (http://geneontology.org/docs/go-enrichment-analysis/ and http://pantherdb.org). Enriched biological processes with a false discovery rate (FDR) corrected *p*-value under 0.05 are reported.

### Colocalization

We performed a colocalization analysis similar to the probabilistic method for integrating GWAS and eQTL data presented in eCAVIAR^86^. We used expression data from the EMBL-EBI eQTL Catalogue (https://www.ebi.ac.uk/eqtl/) for 24 tissues/cell types as fine-mapped previously by Alasoo and Keriamov (https://kauralasoo.github.io)^87^. We calculate the causal posterior probability (CLPP) similar to Hormozdiari et al.^86^. In addition, we compute another colocalization metric, the causal posterior agreement (CLPA), that is independent of the size of the credible sets. For variants i intersecting in phenotype 1 (p_1_) and phenotype 2 (p_2_), where vector x contains the posterior inclusion probability for p_1_ and vector y for p_2_, CLPA_*k*_ = Σ_*i*∈CS*k*_min(x_*i*_,y_*i*_) if i is contained in p_1_ and p_2_ (0 otherwise). CLPP represents the probability that the same locus is causal in two studies and CLPA represents the agreement of fine-mapping results between two studies. We employed a causal posterior probability cutoff of ≥ 0.1%.

### Annotation of genetic loci based on previous studies

We reviewed genes in immediate proximity with the lead variants from the GWAS meta-analysis (nearest, 2^nd^ nearest and 3^rd^ nearest protein-coding genes) for previous reports on biological function and disease associations (Supplementary Table 1). The biological functions of proteins were evaluated based on UniProt and followed up with literature review. ClinVar and GWAS Catalog were queried for associations with severe skeletal or dental diseases. Mouse knockout data were queried using PubMed searches and available International Mouse Phenotyping Consortium (IMPC) data (https://www.mousephenotype.org; accessed April 25 2022) using the consortium’s recommended *p*-value threshold of 0.0001 to correct for multiple comparisons^88,89^.

### Immunohistochemistry

Pregnant CD1 mice were obtained from Charles River Laboratories, Wilmington, CA, and were housed at 20–22 °C ambient temperature with 30–70% humidity, a 12-hour light/dark cycle, and food and water available ad libitum. All procedures were approved by the Administrative Panel on Laboratory Animal Care (APLAC) at Stanford University according to the National Institutes of Health guidelines for animal care (protocol #33998). Postnatal day 2 (P2) and 6 (P6) mice of both sexes were sacrificed, decapitated, and their temporal bones extracted. The ossicles were removed with fine forceps, the round window membrane was punctured, and the cochlea was flushed with 4% paraformaldehyde (PFA) fixative, and decalcified in 0.12 M EDTA for 24 h. Postnatal day 12 (P12) and adult, 3 month old mice were anesthetized with Ketamine (100 mg/kg) and Xylazine (10 mg/kg) and perfused intracardially with 4% PFA. Their temporal bones were collected, and inner ears were perfused with 4% PFA as described above. After decalcified in 0.12 M EDTA for 72 h, cochleae of all ages were cryoprotected in 30% sucrose, embedded in optimal cutting temperature (O.C.T.) compound, snap frozen on dry ice, and stored at −80 °C before further processing. O.C.T. blocks were sectioned on a Leica cryostat at 12 µm sections, mounted on glass slides, and stored at −20 °C. For immunofluorescent staining, tissue sections were washed with PBS three times, blocked with 5% NHS and 0.3% TX-100 in PBS for 1 h, and immunostained overnight at room temperature with the following primary antibodies diluted in 1% NHS and 0.3% TX-100: rabbit polyclonal anti-MEPE (Kerafast, Boston, MA, #ENH086-FP, 1:400;) and rabbit monoclonal RUNX2 (Cell Signaling Technology, Danvers, MA, clone D1L7F, #12556, 1:200)^90^. Sections were then washed in PBS and incubated with goat anti-rabbit Alexa Fluor 488 antibodies (Thermo Fisher Scientific, Waltham, MA, #A-11008, 1:500). The nuclei were counterstained with DAPI solution (Thermo Fisher Scientific, Waltham, MA, #62248, 1: 10000). Sections were imaged on a Zeiss LSM 880 confocal microscope (Jena, Germany). For immunoperoxidase staining, sections were prepared by the Stanford animal histopathology core’s standard staining process. The sections were washed and blocked with avidin, biotin, and 3% goat serum in TBS-Tween. Slides were incubated overnight with primary antibodies, endogenous peroxidases were inactivated with 3% hydrogen peroxide solution, incubated with goat anti-rabbit IgG (Abcam, Cambridge, UK, #ab64256, 5 µg/mL), washed, and incubated with horseradish peroxidase-conjugated streptavidin (Thermo Fisher Scientific, Waltham, MA, # N100, 1:1000) for signal amplification, and stained with DAB chromogen and hematoxylin. Slides were imaged on a Zeiss (Jena, Germany) AxioImager widefield microscope. As a negative control, primary antibodies were omitted from the staining protocol, resulting in no specific signal. As a positive control, previously validated, unrelated primary antibodies gave rise to different specific patterns of expression. Representative images from *N* = 3 animals of each age group are presented.

### Reporting summary

Further information on research design is available in the Nature Research Reporting Summary linked to this article.

## Supplementary information

Supplementary Information

Description of Additional Supplementary Files

Supplementary Data 1-26

Reporting Summary

## Data Availability

The meta-analysis summary statistics generated in this study have been deposited in the NHGRI-EBI GWAS Catalog under the accession code GCST90129575. Cohort-level summary statistics from FinnGen are publicly available (https://r6.finngen.fi/pheno/H8_OTOSCLE). Individual-level genotypes and register data from FinnGen participants can be accessed by approved researchers via the Fingenious portal (https://site.fingenious.fi/en/) hosted by the Finnish Biobank Cooperative FinBB (https://finbb.fi/en/). Data from the UK Biobank are available by application to all bone fine researchers in the public interest (https://www.ukbiobank.ac.uk/enable-your-research/apply-for-access). The individual-level Estonian Biobank data are available under restricted access administered by the Estonian Genome Center of the University of Tartu (EGCUT) in accordance with the regulations of the Estonian Human Genes Research Act; access can be obtained by application at www.biobank.ee.

## Code availability

No new algorithms were generated as part of this study. Previously published software were used to generate the analyses as detailed in the Methods section, and exact commands with parameters can be made available upon request.
