# Supplementary material for: Genome-wide screen of otosclerosis in population biobanks: 27 loci and shared associations with skeletal structure
Source: Nat Commun. 2023 Jan 18;14:157. doi: 10.1038/s41467-022-32936-3 (PMC9849444; doi:10.1038/s41467-022-32936-3)
Supplement: Supplementary file 1 — Supplementary Information [file 41467_2022_32936_MOESM1_ESM.pdf]

Supplementary Information

Table of contents

Supplementary Table 2
Supplementary Figures 4
Supplementary Figure 1. Cohort-specific Manhattan plots 4
Supplementary Figure 2. SNP-based heritability of otosclerosis in each cohort 6
Supplementary Figure 3. LocusZoom plots for significant loci in the meta-analysis 7
Supplementary Figure 4. Correlation analysis of lead variant associations for otosclerosis and selected traits in FinnGen 12
Supplementary Figure 5. Meta-analysis LocusZoom plots for candidate gene regions 14
Supplementary Figure 6. Additional genome-wide association studies of otosclerosis in FinnGen 16
Supplementary Figure 7. QQ-plots from post hoc permutation analyses with MAGMA 19
FinnGen Consortium Members 21
Supplementary References 35

Supplementary Table

Rsid	Chromosome and position	Nearest Gene	Biological role(s) of proteins coded by nearest genes	Severe skeletal diseases associated with nearest genes	Hearing or skeletal phenotypes in mouse knockout models
rs11683921	2:111714056	*ANAPC1*	*ANAPC1*: *Encodes sc*affold subunit of the anaphase-promoting complex	*ANAPC1*: Rothmund-Thomson Syndrome Type 1 (skeletal and dental abnormalities and short stature)	
rs4917	3:186619924	*AHSG*	*AHSG:* Binds to TGFβ1 and BMPs. Regulator of mineralization through inhibition of calcium phospate precipitation. AHSG knockout mice exhibit increased bone formation with age and ectopic bone formation in response to TGFβ1/BMP signalling.		AHSG: High frequency hearing loss
rs181831514	4:87901594	*MEPE*	*MEPE:* Candidate gene for otosclerosis. Regulator of osteoclast differentiation and mineralization. *IBSP* (3rd nearest gene): Structural protein of bone matrix	*MEPE*: Hereditary congenital facial paresis	
rs13192457	6:44887654	*SUPT3H*	*RUNX2* (2nd nearest gene, overlapping association signal): Transcription factor, regulator of osteoblast and chondrocyte differentiation. *RUNX2* and *SUPT3H* show synteny across several species and the SUPT3H promoter is a potential regulator of the bone-specific Runx2-P1 promoter.	*RUNX2*: Metaphyseal dysplasia with maxillary hypoplasia and brachydactyly, cleidocranial dysostosis	RUNX2: Hearing loss
rs4464751	6:73707844	*CD109*	*CD109*: TGF-β co-receptor, negative regulator of TGF-β signalling		
rs77249084	8:116560300	*EIF3H*		Rs13279799: Associated with otosclerosis and ossification of the posterior longitudinal ligament of the spine	
rs4877080	9:89398813	*SEMA4D*	*SEMA4D*: Expressed in osteoclasts, inhibits bone formation		SEMA4D: Low bone mineral content
rs12270054	11:65555077	*LTBP3*	*LTBP3*: Latent Transforming Growth Factor Beta Binding Protein 3 (LTBP3), a component of the extracellular matrix. Regulates TGFβ activity via extracellular binding.	*LTBP3:* Acromicric dysplasia, Brachyolmia-amelogenesis imperfecta syndrome, Geleophysic dysplasia.	
rs73172296	13:42525753	*TNFSF11*	TNFSF11: Osteoclast differentiation and activation factor	TNFSF11: Osteopetrosis	
rs7995158	13:110459370	*COL4A2 (COL4A2-AS2)*	*COL4A2:* Alpha-2 chain of basement membrane collagen.		
rs10592836	14:103471816	*MARK3*	*MARK3:* Potential regulator of bone mineral density. *CKB* (2nd nearest gene, overlapping association): Regulator of the bone-resorbing function of osteoclasts.		
rs2118612	15:67108152	*SMAD3*	*SMAD3*: Downstream transcription factor in the TGFβ1 signalling pathway	*SMAD3*: Aneurysms-osteoarthritis syndrome	
rs67284550	16:1480948	*PTX4*	*CLCN7* (2nd nearest gene, nearest gene to fine-mapped credible set): Regulator of osteoblast function. *TELO2* (3rd nearest gene): Overexpressed in bone.	*CLCN7*: Osteopetrosis (several types). *TELO2*: You-Hoover-Fong syndrome	
rs4636903	16:54995451	*IRX5*	*IRX5*: Regulator of mineralization in cranial bones.	*IRX5:* Hamamy syndrome (with features including skeletal abnormalities, short stature, enamel hypoplasia, osteopenia with repeated fracture, and hearing loss)	
rs11868207	17:68679579	*FAM20A*	*FAM20A*: Involvement in biomineralization of teeth	*FAM20A*: Amelogenesis imperfecta type IG	
rs8105161	19:41333726	*TGFB1*	*TGFB1*: Candidate gene for otosclerosis. Essential cytokine for skeletal development and bone remodeling. Regulator of osteoblast and osteoclast cell lineages.	*TGFB1*: Gain-of-function mutations predispose to diaphyseal dysplasia (Camurati-Engelman disease)	
rs6066131	20:46941020	*EYA2*			EYA2: Mild hearing loss
rs6066825	20:48723580	*PREX1*	*PREX1*: Potential driver of bone dissemination in breast cancer		

Supplementary Table 1. Annotation of association loci with biological functions and rare disease associations. In several association loci, genes implicated in bone metabolism are in close proximity with the lead variant. Nearest protein coding genes to the lead variants are highlighted in bold. The biological functions of proteins were evaluated based on UniProt and followed up with literature review. ClinVar and GWAS Catalog were queried for associations with severe skeletal or dental diseases. Mouse knockout data were queried using available International Mouse Phenotyping Consortium (IMPC) data and PubMed searches. References to individual studies are listed in Supplementary References.

Supplementary Figures

Supplementary Figure 1. Cohort-specific Manhattan plots

a) FinnGen

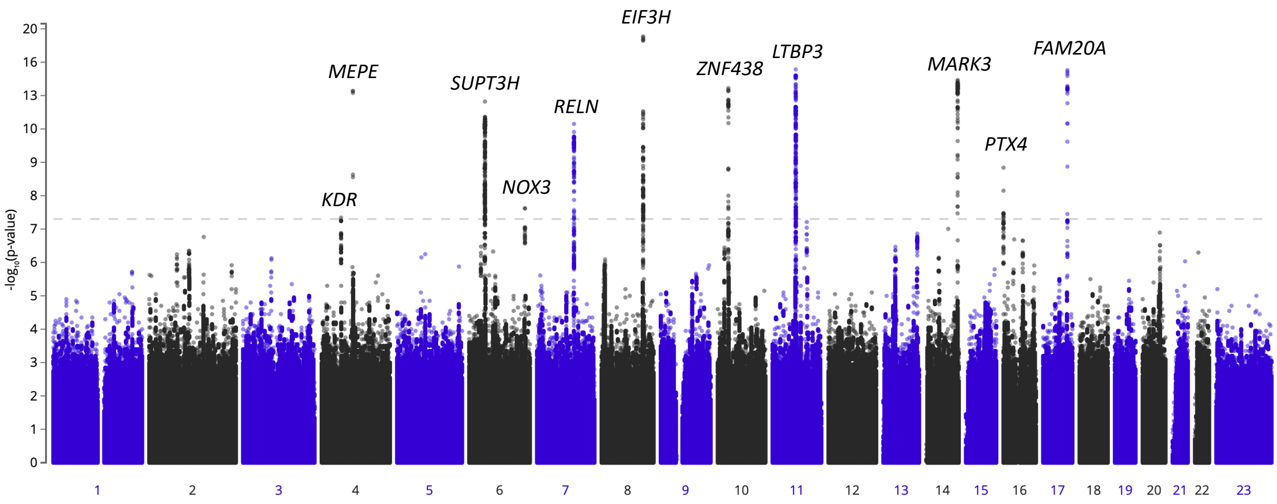

b) EstBB

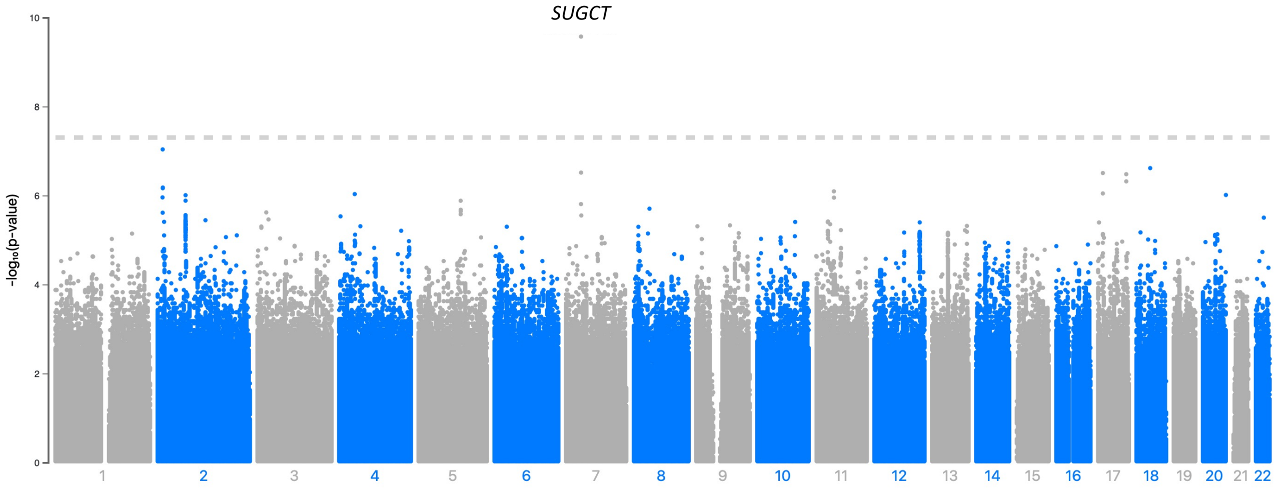

c) UKBB

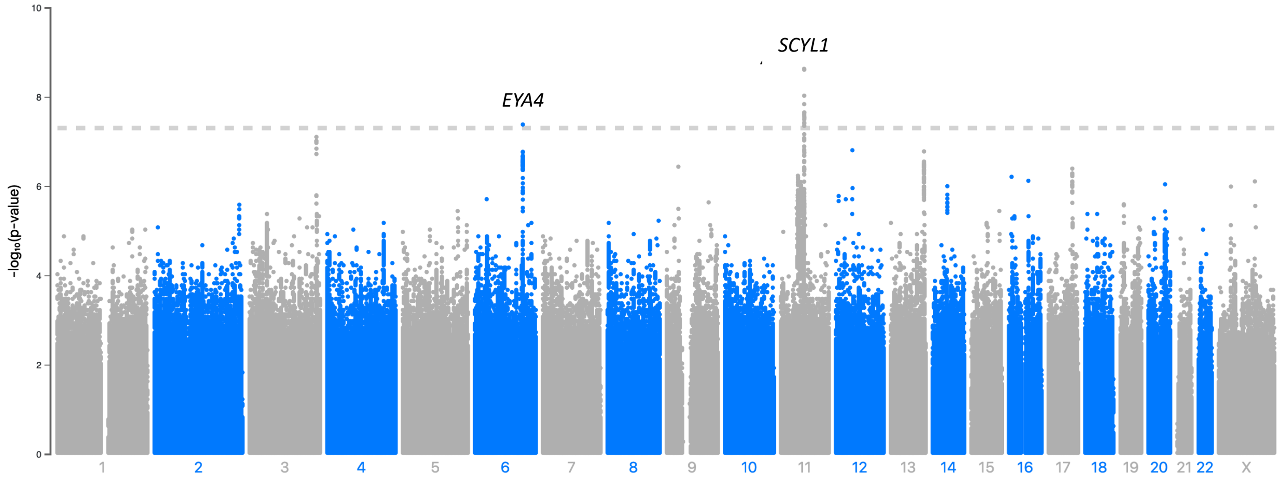

Manhattan plots are presented for the case-control GWAS of otosclerosis in a) FinnGen (1,563 cases and 249,281 controls), b) EstBB (985 cases and 196,516 controls), and c) UKBB (956 cases and 415,401 controls). GWAS for each individual study cohort was performed using a generalized mixed model with the saddlepoint approximation using SAIGE v0.20, using a kinship matrix as a random effect and covariates as fixed effects. Variants with a minor allele frequency > 0.1% and imputation INFO score ≥ 0.7 were included in the analysis. A Bonferroni-corrected two-sided *p*-value threshold of $5x10^{-8}$ (dashed line) was used to account for multiple comparisons. Colours are used to visually separate chromosomes.

Supplementary Figure 2. SNP-based heritability of otosclerosis in each cohort

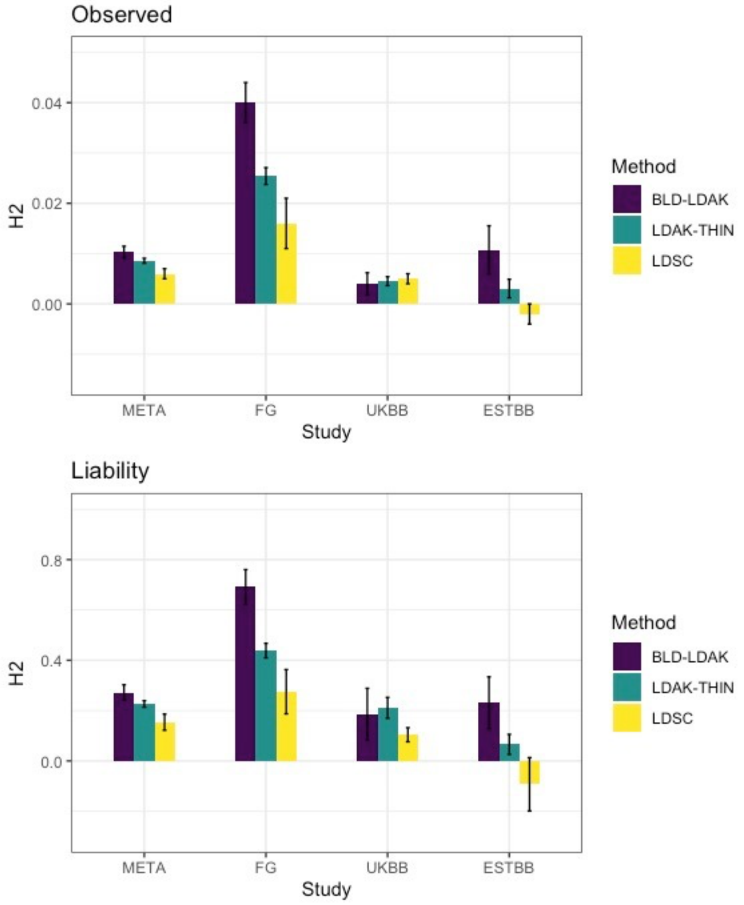

Approximate estimates for the narrow sense heritability of otosclerosis in each cohort and the subsequent meta-analysis were obtained and compared using three different summary statistics based methods: 1) LD Score Regression (LDSC), and 2) BLK-LDAK and 3) LDAK-Thin of the SumHer software. We used summary statistics separately from the meta-analysis and each cohort, restricting the analyses to variants present in HapMap3. For the BLK-LDAK and LDAK-Thin models, we used the European tagging files based on UKBB (http://dougspeed.com/pre-computed-tagging-files/), and for LDSC we used LD Scores computed using 1000 Genomes European data (https://data.broadinstitute.org/alkesgroup/LDSCORE/eur_w_ld_chr.tar.bz2). For the liability transformations, a population prevalence approximation of 0.3% was used. Heritability estimates are presented as bars and the standard errors are indicated for each heritability estimate with error bars. A total of 250,844 FinnGen samples, 197,501 Estonian Biobank samples and 416,357 UK Biobank samples were used for heritability estimation. The precise heritability estimates, their standard errors and corresponding Z-scores are presented in Supplementary Data 5.

Supplementary Figure 3. LocusZoom plots for significant loci in the meta-analysis

***EIF3H* locus**

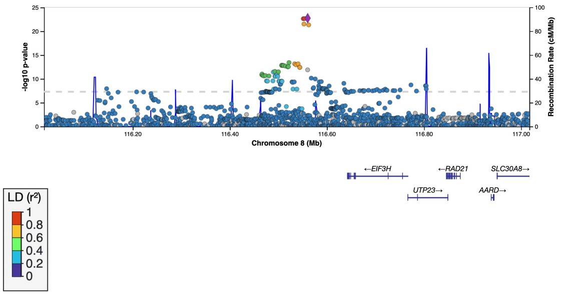

***FAM20A (LINC01482)* locus**

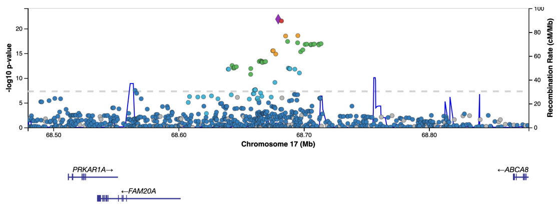

***LTBP3* locus**

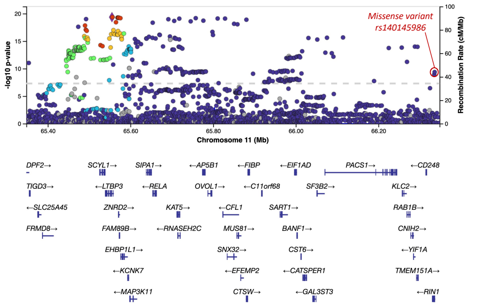

***MARK3* locus**

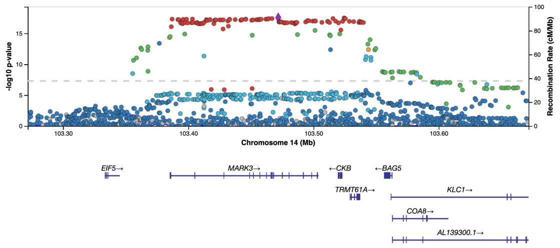

***ZNF438* locus**

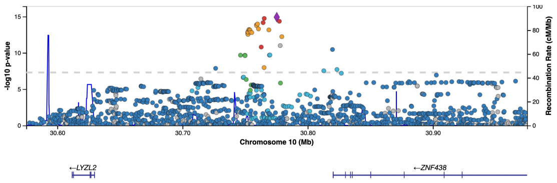

***COL4A2 (COL4A2-AS2)* locus**

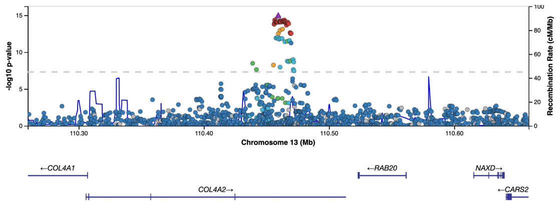

***RELN* locus**

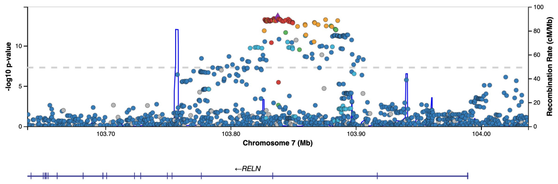

***IP6K3* locus**

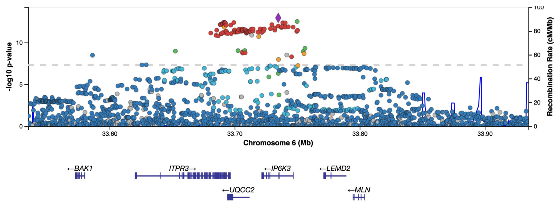

***MEPE* locus**

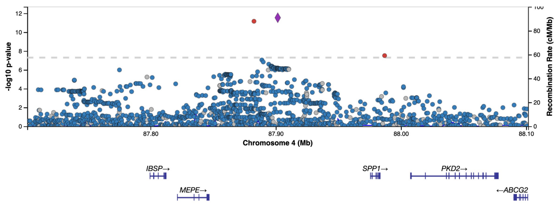

***AHSG* locus**

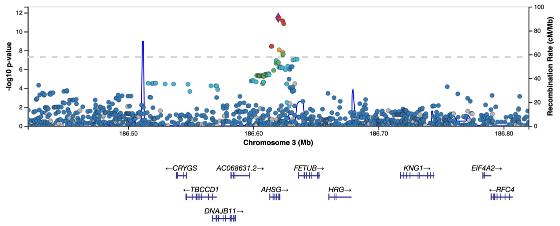

***SUPT3H* locus**

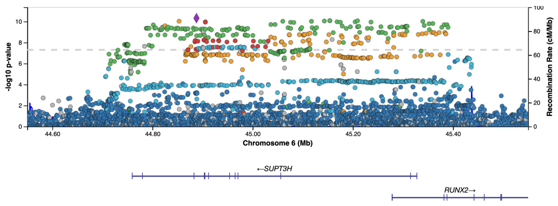

***IRX5* locus**

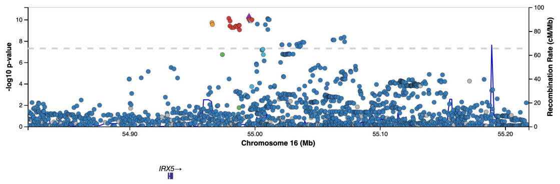

***PTX4* locus**

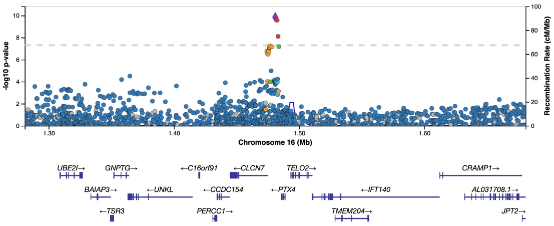

***ANAPC1* locus**

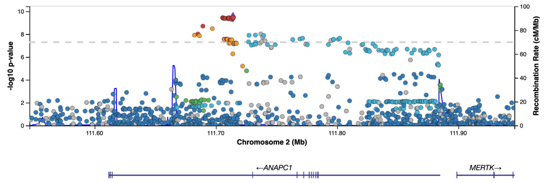

***MAML2* locus**

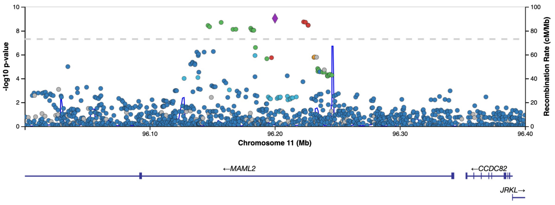

***TNFSF11* locus**

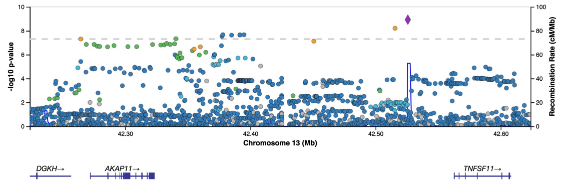

***CLDN23* locus**

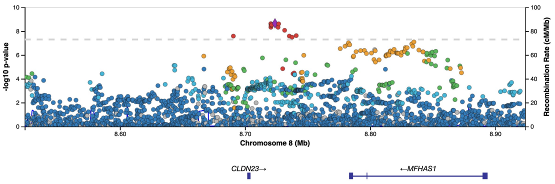

***KCNRG (DLEU1)* locus**

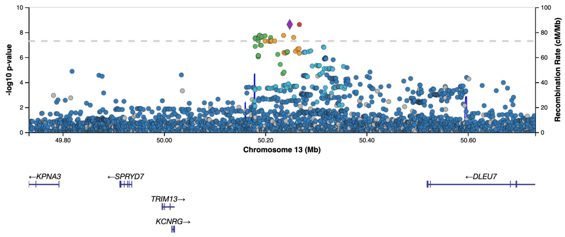

***SEMA4D* locus**

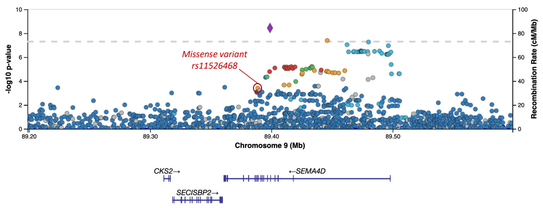

***ZC3H11B* locus**

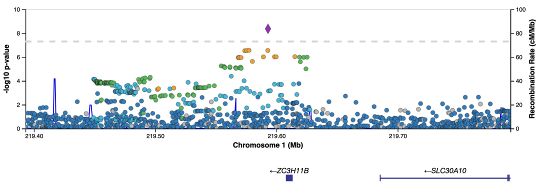

***PREX1* locus**

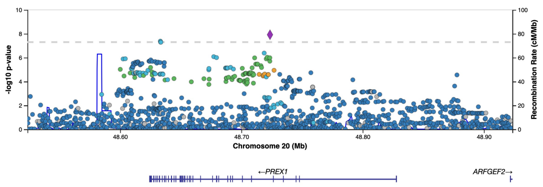

***DNMT1* locus**

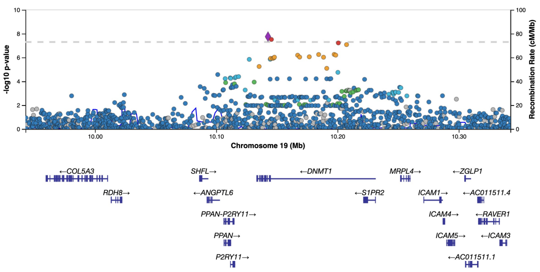

***TGFB1* locus**

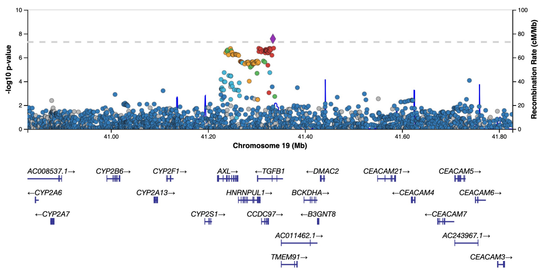

***SMAD3* locus**

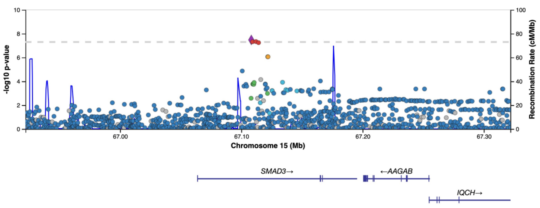

CD109 locus

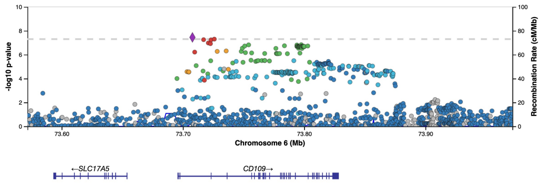

STX17 (STX17-AS1) locus

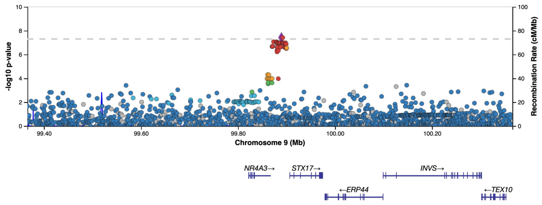

EYA2 locus

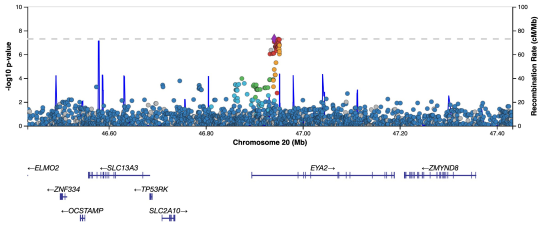

GWAS for each individual study cohort was performed using a generalized mixed model with the saddlepoint approximation using SAIGE v0.20, using a kinship matrix as a random effect and covariates as fixed effects. A fixed-effect meta-analysis of effect estimates from the three cohorts was then performed including a total of 3,504 cases and 861,198 controls. Twenty-seven loci reached genome-wide significance in the meta-analysis (two-sided p-value < $5x10^{-8}$ to account for multiple comparisons, marked by the dashed line). LocusZoom plots for each significant locus are shown in the same order as in Table 2, and the exact *p*-values corresponding to lead variants in each locus are presented in Table 2. In the LocusZoom plots, variant positions are indicated on the x-axis and $-\log_{10}$(*p*-value) is presented on the y-axis for each variant. The included variants were present in at least two cohorts with a cross-cohort minor allele frequency > 0.1% and imputation INFO score ≥ 0.7. The loci are annotated by the names of the coding genes nearest to the lead variants. Linkage disequilibrium is shown in colour scale with the lead variants as reference, based on publicly available European genome data from the 1000 Genomes Project.

Supplementary Figure 4. Correlation analysis of lead variant associations for otosclerosis and selected traits in FinnGen

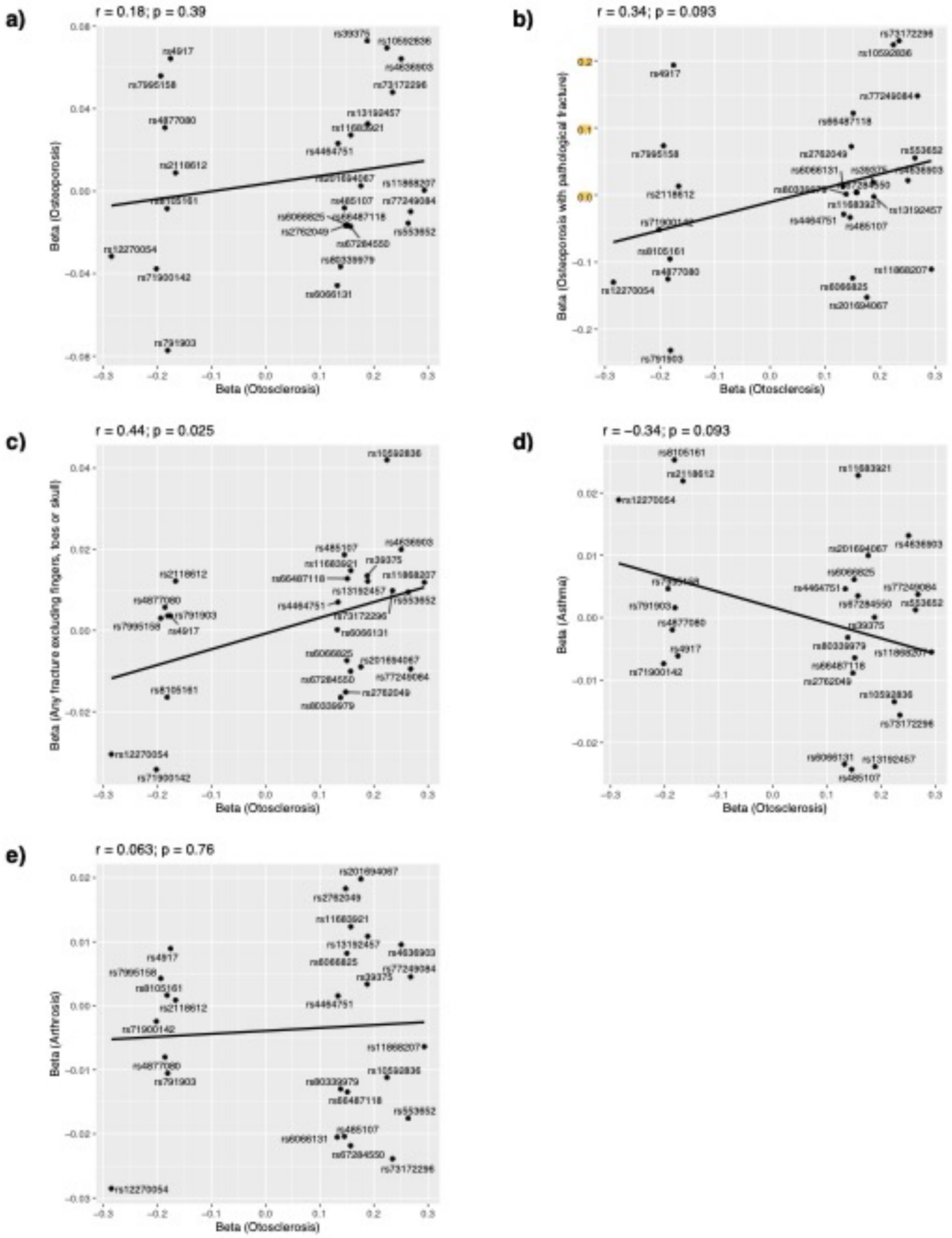

Lead variants from the 27 significant otosclerosis GWAS meta-analysis loci were analyzed with respect to their association with selected traits in FinnGen (prioritized based on the earlier phenome-wide association study). The beta estimate for otosclerosis is represented on the x-axis, and the beta estimate for the trait under comparison is represented on the y-axis. Included traits were a) osteoporosis, b) osteoporosis with pathological fracture, c) any fracture excluding fracture of fingers, toes or skull, d) asthma, e) arthrosis. Pearson's correlation coefficients and corresponding *p*-values (using the t-distribution) were computed for all comparisons. A two-sided *p*-value threshold of 0.01 was used to account for multiple comparisons.

Supplementary Figure 5. Meta-analysis LocusZoom plots for candidate gene regions

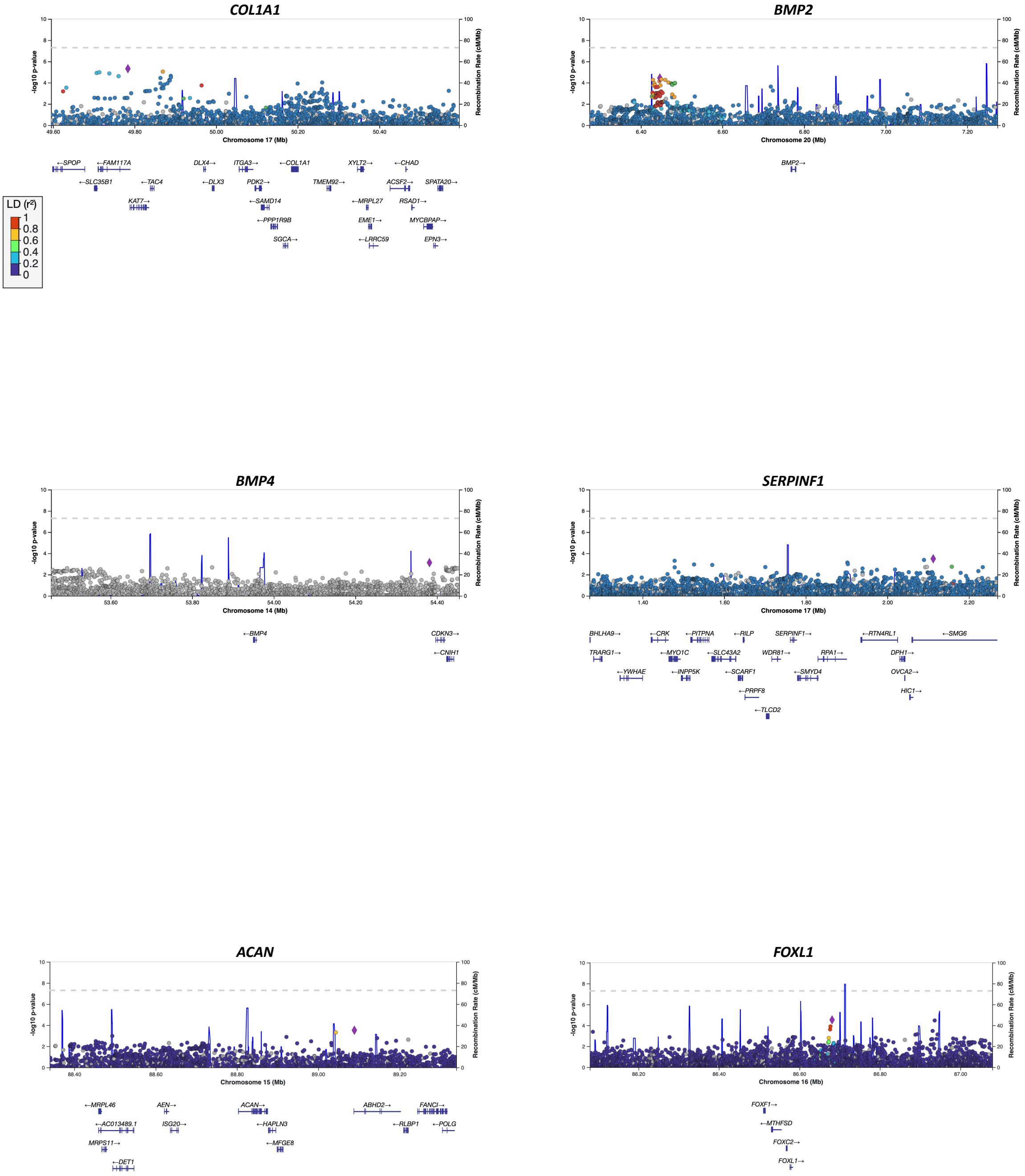

GWAS for each individual study cohort was performed using a generalized mixed model with the saddlepoint approximation using SAIGE v0.20, using a kinship matrix as a random effect and covariates as fixed effects. A fixed-effect meta-analysis of effect estimates from the three cohorts was then performed including a total of 3,504 cases and 861,198 controls. LocusZoom plots are presented for the regions of four candidate genes based on previous studies (*COL1A1*, *BMP2*, *BMP4*, and *SERPINF1*). In the LocusZoom plots, variant positions are indicated on the x-axis and $-\log_{10}$(*p*-value) is presented on the y-axis for each variant. The included variants were present in at least two cohorts with a cross-cohort minor allele frequency > 0.1% and imputation INFO score ≥ 0.7. The loci are annotated by the names of the coding genes nearest to the lead variants. Linkage disequilibrium is shown in colour scale with the lead variants as reference, based on publicly available European genome data from the 1000 Genomes Project. Odds ratios and *p*-values for candidate variants are also presented in Supplementary Data 20.

Supplementary Figure 6. Additional genome-wide association studies of otosclerosis in FinnGen

Women only

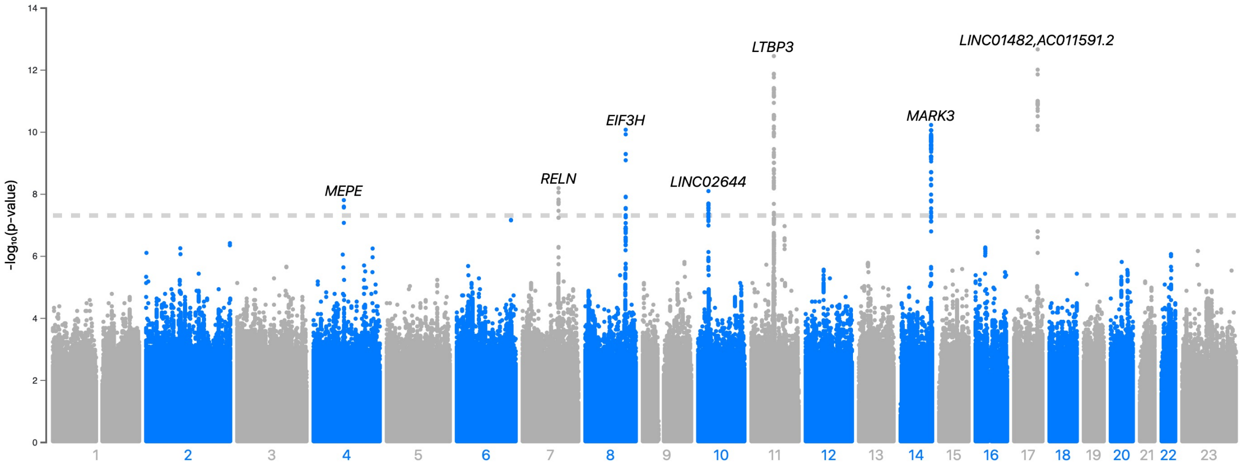

Men only

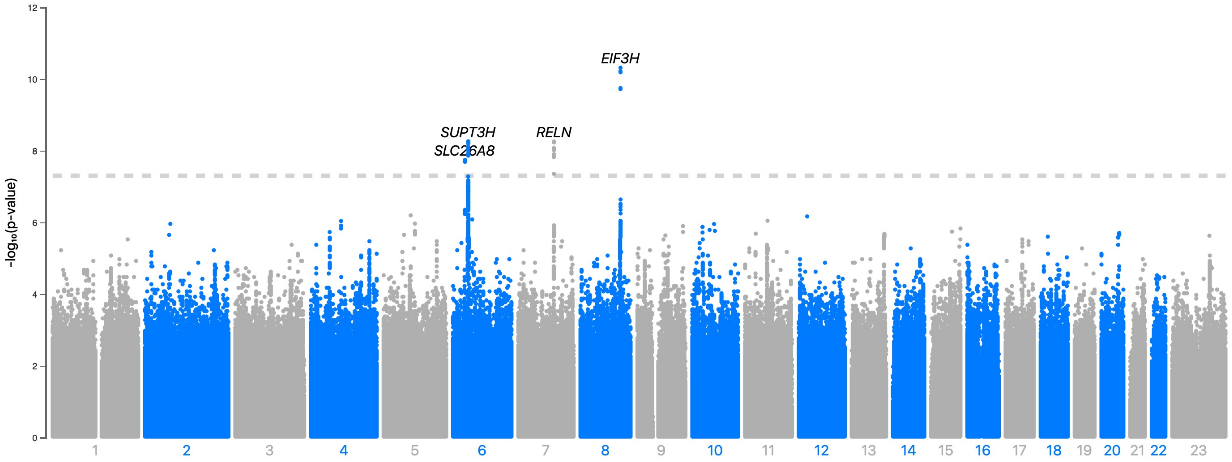

Controls limited to individuals aged > 65

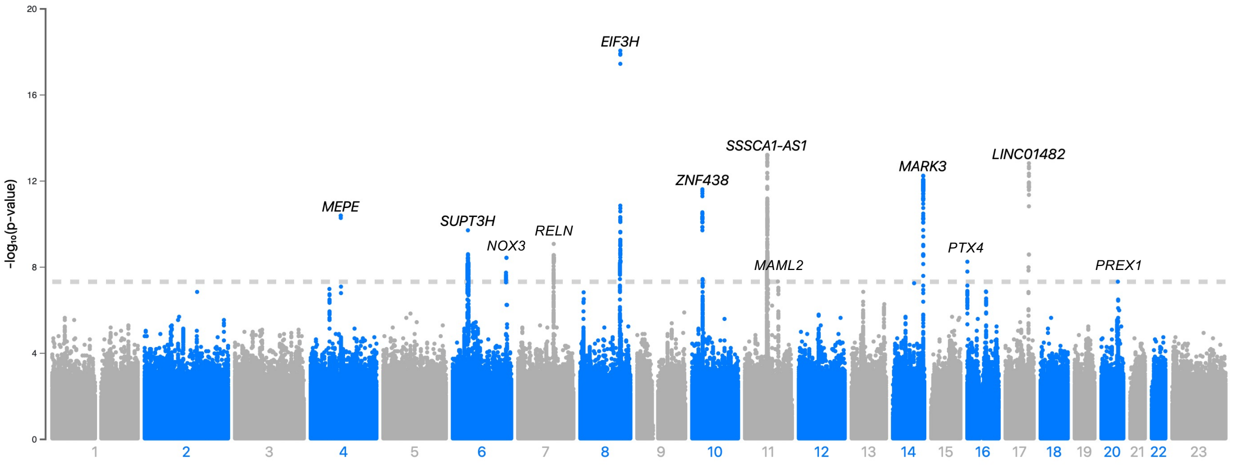

Controls limited to individuals without hearing loss

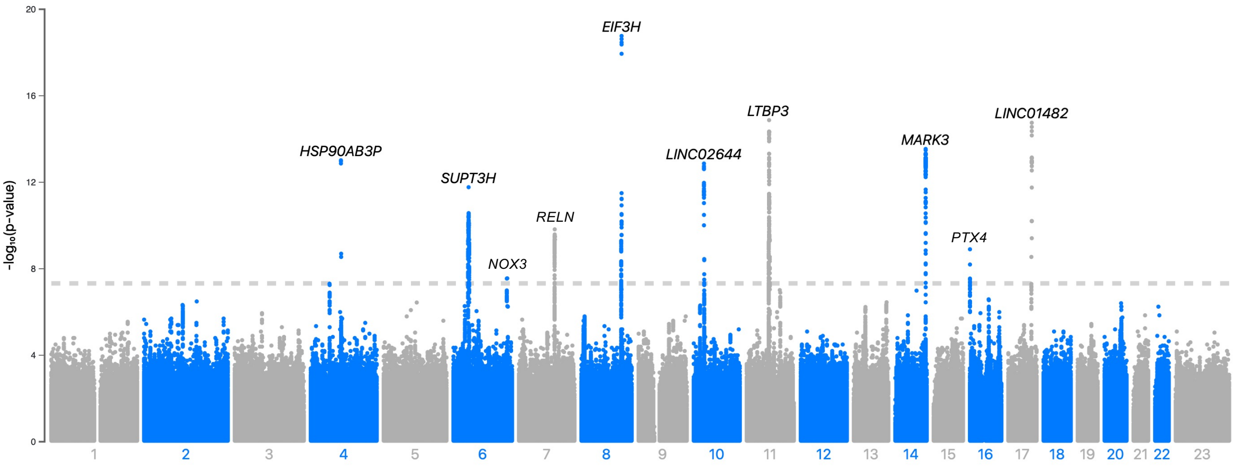

Controls limited to individuals aged > 65 without hearing loss

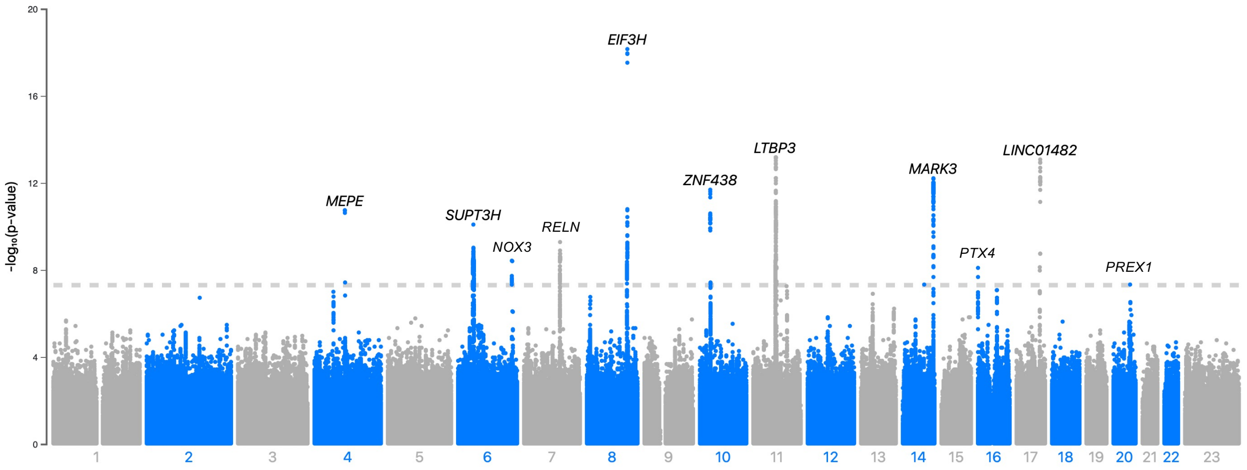

Additional GWAS were conducted in FinnGen for women (1002 cases and 140,222 controls) and men (561 cases and 109,059 controls). GWAS were also performed comparing all 1,563 cases with controls filtered to include only individuals over age 75 (110,166 controls), individuals without a diagnosis of hearing loss (231,502 controls) and individuals over age 75 without a diagnosis of hearing loss (96,564 controls). GWAS were performed using a generalized mixed model with the saddlepoint approximation using SAIGE v0.20, using a kinship matrix as a random effect and covariates as fixed effects. A Bonferroni-corrected two-sided genome-wide *p*-value threshold of $5x10^{-8}$ was used to account for multiple comparisons. Colours are used to visually separate chromosomes.

Supplementary Figure 7. QQ-plots from post hoc permutation analyses with MAGMA

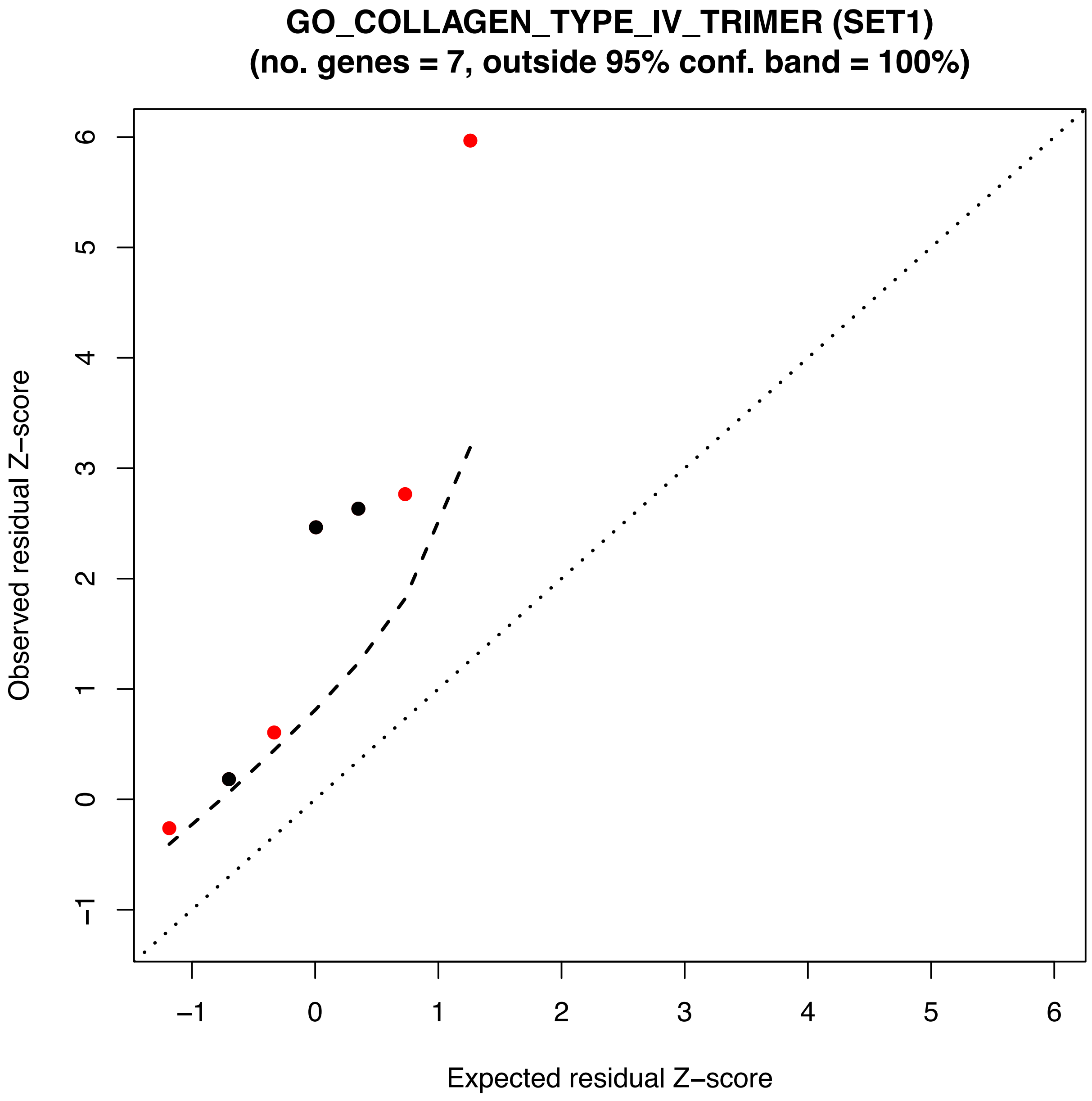

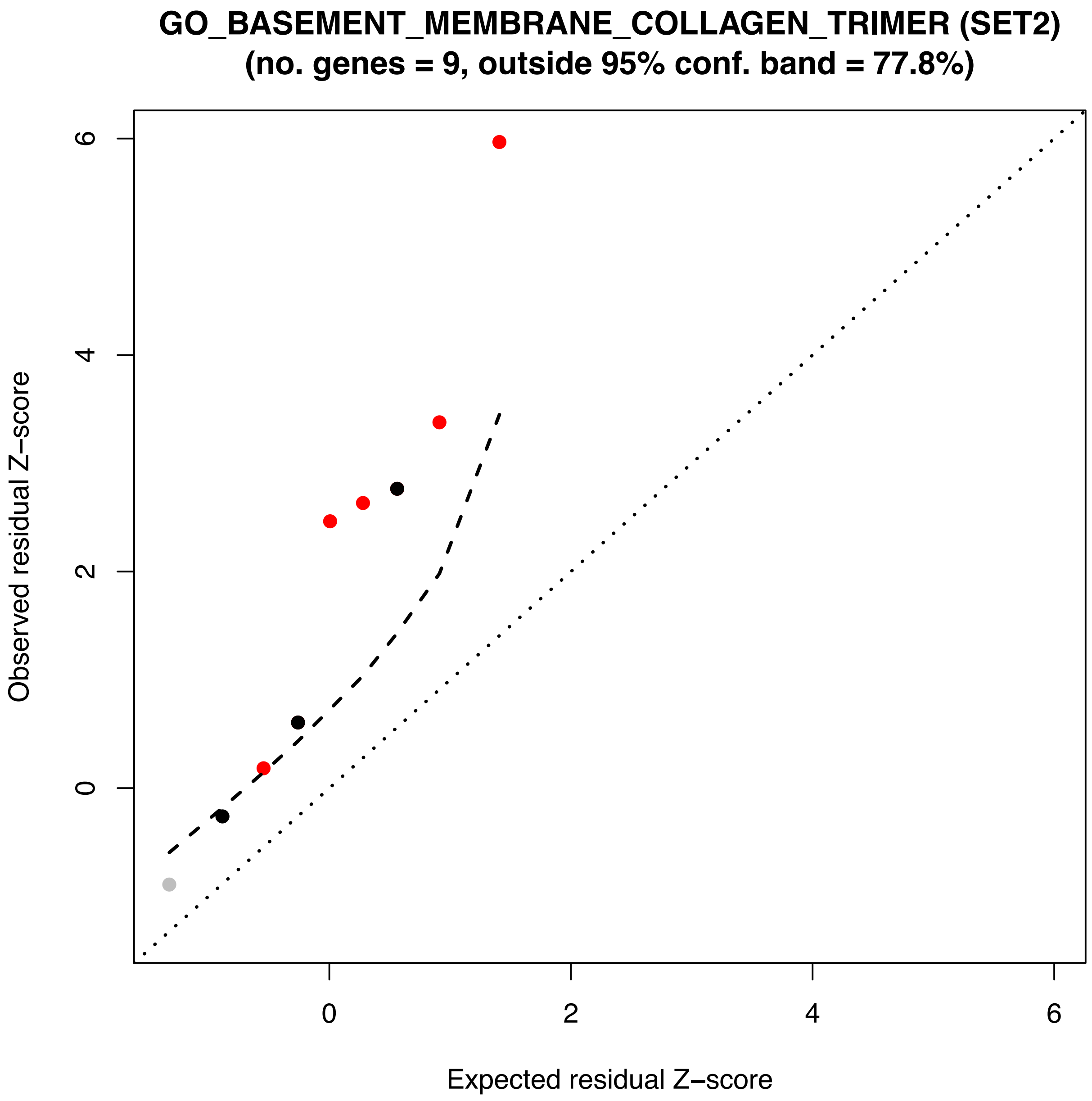

Based on the results from the gene-based analysis of the meta-analysis summary statistics, a competitive gene set based analysis was performed using 10,182 GO term based gene sets downloaded from the Molecular Signature Database v7.1, using a Bonferroni-corrected *p*-value threshold (α = 0.05/10,182). Set-specific QQ plots were produced by permutation analysis to evaluate for the influence of outliers.

FinnGen Consortium Members

Full Name	Affiliation
Aarno Palotie	Institute for Molecular Medicine Finland (FIMM), HiLIFE, University of Helsinki, Helsinki, Finland; Broad Institute of MIT and Harvard; Massachusetts General Hospital
Mark Daly	Institute for Molecular Medicine Finland (FIMM), HiLIFE, University of Helsinki, Helsinki, Finland; Broad Institute of MIT and Harvard; Massachusetts General Hospital
Bridget Riley-Gills	Abbvie, Chicago, IL, United States
Howard Jacob	Abbvie, Chicago, IL, United States
Dirk Paul	Astra Zeneca, Cambridge, United Kingdom
Athena Matakidou	Astra Zeneca, Cambridge, United Kingdom
Adam Platt	Astra Zeneca, Cambridge, United Kingdom
Heiko Runz	Biogen, Cambridge, MA, United States
Sally John	Biogen, Cambridge, MA, United States
George Okafo	Boehringer Ingelheim, Ingelheim am Rhein, Germany
Nathan Lawless	Boehringer Ingelheim, Ingelheim am Rhein, Germany
Heli Salminen-Mankonen	Boehringer Ingelheim, Ingelheim am Rhein, Germany
Robert Plenge	Bristol Myers Squibb, New York, NY, United States
Joseph Maranville	Bristol Myers Squibb, New York, NY, United States
Mark McCarthy	Genentech, San Francisco, CA, United States
Julie Hunkapiller	Genentech, San Francisco, CA, United States
Margaret G. Ehm	GlaxoSmithKline, Collegeville, PA, United States
Kirsi Auro	GlaxoSmithKline, Espoo, Finland
Simonne Longerich	Merck, Kenilworth, NJ, United States
Caroline Fox	Merck, Kenilworth, NJ, United States
Anders Mälarstig	Pfizer, New York, NY, United States
Katherine Klinger	Translational Sciences, Sanofi R&D, Framingham, MA, USA
Deepak Raipal	Translational Sciences, Sanofi R&D, Framingham, MA, USA
Eric Green	Maze Therapeutics, San Francisco, CA, United States
Robert Graham	Maze Therapeutics, San Francisco, CA, United States
Robert Yang	Janssen Biotech, Beerse, Belgium
Chris O´Donnell	Novartis Institutes for BioMedical Research, Cambridge, MA, United States
Tomi P. Mäkelä	HiLIFE, University of Helsinki, Finland, Finland
Jaakko Kaprio	Institute for Molecular Medicine Finland (FIMM), HiLIFE, University of Helsinki, Helsinki, Finland
Petri Virolainen	Auria Biobank / University of Turku / Hospital District of Southwest Finland, Turku, Finland

Antti Hakanen	Auria Biobank / University of Turku / Hospital District of Southwest Finland, Turku, Finland
Terhi Kilpi	THL Biobank / Finnish Institute for Health and Welfare (THL), Helsinki, Finland
Markus Perola	THL Biobank / Finnish Institute for Health and Welfare (THL), Helsinki, Finland
Jukka Partanen	Finnish Red Cross Blood Service / Finnish Hematology Registry and Clinical Biobank, Helsinki, Finland
Anne Pitkäranta	Helsinki Biobank / Helsinki University and Hospital District of Helsinki and Uusimaa, Helsinki
Taneli Raivio	Helsinki Biobank / Helsinki University and Hospital District of Helsinki and Uusimaa, Helsinki
Raisa Serpi	Northern Finland Biobank Borealis / University of Oulu / Northern Ostrobothnia Hospital District, Oulu, Finland
Tarja Laitinen	Finnish Clinical Biobank Tampere / University of Tampere / Pirkanmaa Hospital District, Tampere, Finland
Veli-Matti Kosma	Biobank of Eastern Finland / University of Eastern Finland / Northern Savo Hospital District, Kuopio, Finland
Jari Laukkanen	Central Finland Biobank / University of Jyväskylä / Central Finland Health Care District, Jyväskylä, Finland
Marco Hautalahti	FINBB - Finnish biobank cooperative
Outi Tuovila	Business Finland, Helsinki, Finland
Raimo Pakkanen	Business Finland, Helsinki, Finland
Jeffrey Waring	Abbvie, Chicago, IL, United States
Bridget Riley-Gillis	Abbvie, Chicago, IL, United States
Fedik Rahimov	Abbvie, Chicago, IL, United States
Ioanna Tachmazidou	Astra Zeneca, Cambridge, United Kingdom
Chia-Yen Chen	Biogen, Cambridge, MA, United States
Heiko Runz	Biogen, Cambridge, MA, United States
Zhihao Ding	Boehringer Ingelheim, Ingelheim am Rhein, Germany
Marc Jung	Boehringer Ingelheim, Ingelheim am Rhein, Germany
Shameek Biswas	Bristol Myers Squibb, New York, NY, United States
Rion Pendergrass	Genentech, San Francisco, CA, United States
Julie Hunkapiller	Genentech, San Francisco, CA, United States
Margaret G. Ehm	GlaxoSmithKline, Collegeville, PA, United States
David Pulford	GlaxoSmithKline, Stevenage, United Kingdom
Neha Raghavan	Merck, Kenilworth, NJ, United States
Adriana Huertas-Vazquez	Merck, Kenilworth, NJ, United States
Jae-Hoon Sul	Merck, Kenilworth, NJ, United States
Anders Mälarstig	Pfizer, New York, NY, United States
Xinli Hu	Pfizer, New York, NY, United States
Katherine Klinger	Translational Sciences, Sanofi R&D, Framingham, MA, USA
Robert Graham	Maze Therapeutics, San Francisco, CA, United States
Eric Green	Maze Therapeutics, San Francisco, CA, United States

Sahar Mozaffari	Maze Therapeutics, San Francisco, CA, United States
Dawn Waterworth	Janssen Research & Development, LLC, Spring House, PA, United States
Nicole Renaud	Novartis Institutes for BioMedical Research, Cambridge, MA, United States
Ma´en Obeidat	Novartis Institutes for BioMedical Research, Cambridge, MA, United States
Samuli Ripatti	Institute for Molecular Medicine Finland (FIMM), HiLIFE, University of Helsinki, Helsinki, Finland
Johanna Schleutker	Auria Biobank / Univ. of Turku / Hospital District of Southwest Finland, Turku, Finland
Markus Perola	THL Biobank / Finnish Institute for Health and Welfare (THL), Helsinki, Finland
Mikko Arvas	Finnish Red Cross Blood Service / Finnish Hematology Registry and Clinical Biobank, Helsinki, Finland
Olli Carpén	Helsinki Biobank / Helsinki University and Hospital District of Helsinki and Uusimaa, Helsinki
Reetta Hinttala	Northern Finland Biobank Borealis / University of Oulu / Northern Ostrobothnia Hospital District, Oulu, Finland
Johannes Kettunen	Northern Finland Biobank Borealis / University of Oulu / Northern Ostrobothnia Hospital District, Oulu, Finland
Arto Mannermaa	Biobank of Eastern Finland / University of Eastern Finland / Northern Savo Hospital District, Kuopio, Finland
Katriina Aalto-Setälä	Faculty of Medicine and Health Technology, Tampere University, Tampere, Finland
Mika Kähönen	Finnish Clinical Biobank Tampere / University of Tampere / Pirkanmaa Hospital District, Tampere, Finland
Jari Laukkanen	Central Finland Biobank / University of Jyväskylä / Central Finland Health Care District, Jyväskylä, Finland
Johanna Mäkelä	FINBB - Finnish biobank cooperative
Reetta Kälviäinen	Northern Savo Hospital District, Kuopio, Finland
Valtteri Julkunen	Northern Savo Hospital District, Kuopio, Finland
Hilkka Soininen	Northern Savo Hospital District, Kuopio, Finland
Anne Remes	Northern Ostrobothnia Hospital District, Oulu, Finland
Mikko Hiltunen	University of Eastern Finland, Kuopio, Finland
Jukka Peltola	Pirkanmaa Hospital District, Tampere, Finland
Minna Raivio	Hospital District of Helsinki and Uusimaa, Helsinki, Finland
Pentti Tienari	Hospital District of Helsinki and Uusimaa, Helsinki, Finland
Juha Rinne	Hospital District of Southwest Finland, Turku, Finland
Roosa Kallionpää	Hospital District of Southwest Finland, Turku, Finland
Juulia Partanen	Institute for Molecular Medicine Finland, HiLIFE, University of Helsinki, Finland
Ali Abbasi	Abbvie, Chicago, IL, United States
Adam Ziemann	Abbvie, Chicago, IL, United States
Nizar Smaoui	Abbvie, Chicago, IL, United States
Anne Lehtonen	Abbvie, Chicago, IL, United States
Susan Eaton	Biogen, Cambridge, MA, United States
Heiko Runz	Biogen, Cambridge, MA, United States

Sanni Lahdenperä	Biogen, Cambridge, MA, United States
Shameek Biswas	Bristol Myers Squibb, New York, NY, United States
Julie Hunkapiller	Genentech, San Francisco, CA, United States
Natalie Bowers	Genentech, San Francisco, CA, United States
Edmond Teng	Genentech, San Francisco, CA, United States
Rion Pendergrass	Genentech, San Francisco, CA, United States
Fanli Xu	GlaxoSmithKline, Brentford, United Kingdom
David Pulford	GlaxoSmithKline, Stevenage, United Kingdom
Kirsi Auro	GlaxoSmithKline, Espoo, Finland
Laura Addis	GlaxoSmithKline, Brentford, United Kingdom
John Eicher	GlaxoSmithKline, Brentford, United Kingdom
Qingqin S Li	Janssen Research & Development, LLC, Titusville, NJ 08560, United States
Karen He	Janssen Research & Development, LLC, Spring House, PA, United States
Ekaterina Khramtsova	Janssen Research & Development, LLC, Spring House, PA, United States
Neha Raghavan	Merck, Kenilworth, NJ, United States
Martti Färkkilä	Hospital District of Helsinki and Uusimaa, Helsinki, Finland
Jukka Koskela	Hospital District of Helsinki and Uusimaa, Helsinki, Finland
Sampsa Pikkarainen	Hospital District of Helsinki and Uusimaa, Helsinki, Finland
Airi Jussila	Pirkanmaa Hospital District, Tampere, Finland
Katri Kaukinen	Pirkanmaa Hospital District, Tampere, Finland
Timo Blomster	Northern Ostrobothnia Hospital District, Oulu, Finland
Mikko Kiviniemi	Northern Savo Hospital District, Kuopio, Finland
Markku Voutilainen	Hospital District of Southwest Finland, Turku, Finland
Mark Daly	Institute for Molecular Medicine, Finland (FIMM), HiLIFE, University of Helsinki, Helsinki, Finland; Broad Institute of MIT and Harvard; Massachusetts General Hospital
Ali Abbasi	Abbvie, Chicago, IL, United States
Jeffrey Waring	Abbvie, Chicago, IL, United States
Nizar Smaoui	Abbvie, Chicago, IL, United States
Fedik Rahimov	Abbvie, Chicago, IL, United States
Anne Lehtonen	Abbvie, Chicago, IL, United States
Tim Lu	Genentech, San Francisco, CA, United States
Natalie Bowers	Genentech, San Francisco, CA, United States
Rion Pendergrass	Genentech, San Francisco, CA, United States
Linda McCarthy	GlaxoSmithKline, Brentford, United Kingdom
Amy Hart	Janssen Research & Development, LLC, Spring House, PA, United States
Meijian Guan	Janssen Research & Development, LLC, Spring House, PA, United States
Jason Miller	Merck, Kenilworth, NJ, United States
Kirsi Kalpala	Pfizer, New York, NY, United States

Melissa Miller	Pfizer, New York, NY, United States
Xinli Hu	Pfizer, New York, NY, United States
Kari Eklund	Hospital District of Helsinki and Uusimaa, Helsinki, Finland
Antti Palomäki	Hospital District of Southwest Finland, Turku, Finland
Pia Isomäki	Pirkanmaa Hospital District, Tampere, Finland
Laura Pirilä	Hospital District of Southwest Finland, Turku, Finland
Oili Kaipiainen-Seppänen	Northern Savo Hospital District, Kuopio, Finland
Johanna Huhtakangas	Northern Ostrobothnia Hospital District, Oulu, Finland
Nina Mars	Institute for Molecular Medicine Finland (FIMM), HiLIFE, University of Helsinki, Helsinki, Finland
Ali Abbasi	Abbvie, Chicago, IL, United States
Jeffrey Waring	Abbvie, Chicago, IL, United States
Fedik Rahimov	Abbvie, Chicago, IL, United States
Apinya Lertratanakul	Abbvie, Chicago, IL, United States
Nizar Smaoui	Abbvie, Chicago, IL, United States
Anne Lehtonen	Abbvie, Chicago, IL, United States
Marla Hochfeld	Bristol Myers Squibb, New York, NY, United States
Natalie Bowers	Genentech, San Francisco, CA, United States
Rion Pendergrass	Genentech, San Francisco, CA, United States
Jorge Esparza Gordillo	GlaxoSmithKline, Brentford, United Kingdom
Kirsi Auro	GlaxoSmithKline, Espoo, Finland
Dawn Waterworth	Janssen Research & Development, LLC, Spring House, PA, United States
Fabiana Farias	Merck, Kenilworth, NJ, United States
Kirsi Kalpala	Pfizer, New York, NY, United States
Nan Bing	Pfizer, New York, NY, United States
Xinli Hu	Pfizer, New York, NY, United States
Tarja Laitinen	Pirkanmaa Hospital District, Tampere, Finland
Margit Pelkonen	Northern Savo Hospital District, Kuopio, Finland
Paula Kauppi	Hospital District of Helsinki and Uusimaa, Helsinki, Finland
Hannu Kankaanranta	University of Gothenburg, Gothenburg, Sweden/ Seinäjoki Central Hospital, Seinäjoki, Finland/ Tampere University, Tampere, Finland
Terttu Harju	Northern Ostrobothnia Hospital District, Oulu, Finland
Riitta Lahesmaa	Hospital District of Southwest Finland, Turku, Finland
Nizar Smaoui	Abbvie, Chicago, IL, United States
Glenda Lassi	Astra Zeneca, Cambridge, United Kingdom
Susan Eaton	Biogen, Cambridge, MA, United States
Hubert Chen	Genentech, San Francisco, CA, United States

Rion Pendergrass	Genentech, San Francisco, CA, United States
Natalie Bowers	Genentech, San Francisco, CA, United States
Joanna Betts	GlaxoSmithKline, Brentford, United Kingdom
Kirsi Auro	GlaxoSmithKline, Espoo, Finland
Rajashree Mishra	GlaxoSmithKline, Brentford, United Kingdom
Majd Mouded	Novartis, Basel, Switzerland
Debby Ngo	Novartis, Basel, Switzerland
Teemu Niiranen	Finnish Institute for Health and Welfare (THL), Helsinki, Finland
Felix Vaura	Finnish Institute for Health and Welfare (THL), Helsinki, Finland
Veikko Salomaa	Finnish Institute for Health and Welfare (THL), Helsinki, Finland
Kaj Metsärinne	Hospital District of Southwest Finland, Turku, Finland
Jenni Aittokallio	Hospital District of Southwest Finland, Turku, Finland
Mika Kähönen	Pirkanmaa Hospital District, Tampere, Finland
Jussi Hernesniemi	Pirkanmaa Hospital District, Tampere, Finland
Daniel Gordin	Hospital District of Helsinki and Uusimaa, Helsinki, Finland
Juha Sinisalo	Hospital District of Helsinki and Uusimaa, Helsinki, Finland
Marja-Riitta Taskinen	Hospital District of Helsinki and Uusimaa, Helsinki, Finland
Tiinamaija Tuomi	Hospital District of Helsinki and Uusimaa, Helsinki, Finland
Timo Hiltunen	Hospital District of Helsinki and Uusimaa, Helsinki, Finland
Jari Laukkanen	Central Finland Health Care District, Jyväskylä, Finland
Amanda Elliott	Institute for Molecular Medicine Finland (FIMM), HiLIFE, University of Helsinki, Helsinki, Finland; Broad Institute, Cambridge, MA, USA and Massachusetts General Hospital, Boston, MA, USA
Mary Pat Reeve	Institute for Molecular Medicine Finland (FIMM), HiLIFE, University of Helsinki, Helsinki, Finland
Sanni Ruotsalainen	Institute for Molecular Medicine Finland (FIMM), HiLIFE, University of Helsinki, Helsinki, Finland
Benjamin Challis	Astra Zeneca, Cambridge, United Kingdom
Dirk Paul	Astra Zeneca, Cambridge, United Kingdom
Julie Hunkapiller	Genentech, San Francisco, CA, United States
Natalie Bowers	Genentech, San Francisco, CA, United States
Rion Pendergrass	Genentech, San Francisco, CA, United States
Audrey Chu	GlaxoSmithKline, Brentford, United Kingdom
Kirsi Auro	GlaxoSmithKline, Espoo, Finland
Dermot Reilly	Janssen Research & Development, LLC, Boston, MA, United States
Mike Mendelson	Novartis, Boston, MA, United States
Jaakko Parkkinen	Pfizer, New York, NY, United States
Melissa Miller	Pfizer, New York, NY, United States
Tuomo Meretoja	Hospital District of Helsinki and Uusimaa, Helsinki, Finland
Heikki Joensuu	Hospital District of Helsinki and Uusimaa, Helsinki, Finland
Olli Carpén	Hospital District of Helsinki and Uusimaa, Helsinki, Finland

Johanna Mattson	Hospital District of Helsinki and Uusimaa, Helsinki, Finland
Eveliina Salminen	Hospital District of Helsinki and Uusimaa, Helsinki, Finland
Annika Auranen	Pirkanmaa Hospital District , Tampere, Finland
Peeter Karihtala	Northern Ostrobothnia Hospital District, Oulu, Finland
Päivi Auvinen	Northern Savo Hospital District, Kuopio, Finland
Klaus Elenius	Hospital District of Southwest Finland, Turku, Finland
Johanna Schleutker	Hospital District of Southwest Finland, Turku, Finland
Esa Pitkänen	Institute for Molecular Medicine Finland (FIMM), HiLIFE, University of Helsinki, Helsinki, Finland
Nina Mars	Institute for Molecular Medicine Finland (FIMM), HiLIFE, University of Helsinki, Helsinki, Finland
Mark Daly	Institute for Molecular Medicine Finland (FIMM), HiLIFE, University of Helsinki, Helsinki, Finland; Broad Institute of MIT and Harvard; Massachusetts General Hospital
Relja Popovic	Abbvie, Chicago, IL, United States
Jeffrey Waring	Abbvie, Chicago, IL, United States
Bridget Riley-Gillis	Abbvie, Chicago, IL, United States
Anne Lehtonen	Abbvie, Chicago, IL, United States
Jennifer Schutzman	Genentech, San Francisco, CA, United States
Julie Hunkapiller	Genentech, San Francisco, CA, United States
Natalie Bowers	Genentech, San Francisco, CA, United States
Rion Pendergrass	Genentech, San Francisco, CA, United States
Diptee Kulkarni	GlaxoSmithKline, Brentford, United Kingdom
Kirsi Auro	GlaxoSmithKline, Espoo, Finland
Alessandro Porello	Janssen Research & Development, LLC, Spring House, PA, United States
Andrey Loboda	Merck, Kenilworth, NJ, United States
Heli Lehtonen	Pfizer, New York, NY, United States
Stefan McDonough	Pfizer, New York, NY, United States
Sauli Vuoti	Janssen-Cilag Oy, Espoo, Finland
Kai Kaarniranta	Northern Savo Hospital District, Kuopio, Finland
Joni A Turunen	Helsinki University Hospital and University of Helsinki, Helsinki, Finland; Eye Genetics Group, Folkhälsan Research Center, Helsinki, Finland
Terhi Ollila	Hospital District of Helsinki and Uusimaa, Helsinki, Finland
Hannu Uusitalo	Pirkanmaa Hospital District, Tampere, Finland
Juha Karjalainen	Institute for Molecular Medicine Finland (FIMM), HiLIFE, University of Helsinki, Helsinki, Finland
Esa Pitkänen	Institute for Molecular Medicine Finland (FIMM), HiLIFE, University of Helsinki, Helsinki, Finland
Mengzhen Liu	Abbvie, Chicago, IL, United States
Heiko Runz	Biogen, Cambridge, MA, United States
Stephanie Loomis	Biogen, Cambridge, MA, United States
Erich Strauss	Genentech, San Francisco, CA, United States

Natalie Bowers	Genentech, San Francisco, CA, United States
Hao Chen	Genentech, San Francisco, CA, United States
Rion Pendergrass	Genentech, San Francisco, CA, United States
Kaisa Tasanen	Northern Ostrobothnia Hospital District, Oulu, Finland
Laura Huilaja	Northern Ostrobothnia Hospital District, Oulu, Finland
Katariina Hannula-Jouppi	Hospital District of Helsinki and Uusimaa, Helsinki, Finland
Teea Salmi	Pirkanmaa Hospital District, Tampere, Finland
Sirkku Peltonen	Hospital District of Southwest Finland, Turku, Finland
Leena Koulu	Hospital District of Southwest Finland, Turku, Finland
Nizar Smaoui	Abbvie, Chicago, IL, United States
Fedik Rahimov	Abbvie, Chicago, IL, United States
Anne Lehtonen	Abbvie, Chicago, IL, United States
David Choy	Genentech, San Francisco, CA, United States
Rion Pendergrass	Genentech, San Francisco, CA, United States
Dawn Waterworth	Janssen Research & Development, LLC, Spring House, PA, United States
Kirsi Kalpala	Pfizer, New York, NY, United States
Ying Wu	Pfizer, New York, NY, United States
Pirkko Pussinen	Hospital District of Helsinki and Uusimaa, Helsinki, Finland
Aino Salminen	Hospital District of Helsinki and Uusimaa, Helsinki, Finland
Tuula Salo	Hospital District of Helsinki and Uusimaa, Helsinki, Finland
David Rice	Hospital District of Helsinki and Uusimaa, Helsinki, Finland
Pekka Nieminen	Hospital District of Helsinki and Uusimaa, Helsinki, Finland
Ulla Palotie	Hospital District of Helsinki and Uusimaa, Helsinki, Finland
Maria Siponen	Northern Savo Hospital District, Kuopio, Finland
Liisa Suominen	Northern Savo Hospital District, Kuopio, Finland
Päivi Mäntylä	Northern Savo Hospital District, Kuopio, Finland
Ulvi Gursoy	Hospital District of Southwest Finland, Turku, Finland
Vuokko Anttonen	Northern Ostrobothnia Hospital District, Oulu, Finland
Kirsi Sipilä	Research Unit of Oral Health Sciences Faculty of Medicine, University of Oulu, Oulu, Finland; Medical Research Center, Oulu, Oulu University Hospital and University of Oulu, Oulu, Finland
Rion Pendergrass	Genentech, San Francisco, CA, United States
Hannele Laivuori	Institute for Molecular Medicine Finland (FIMM), HiLIFE, University of Helsinki, Helsinki, Finland
Venla Kurra	Pirkanmaa Hospital District, Tampere, Finland
Laura Kotaniemi-Talonen	Pirkanmaa Hospital District, Tampere, Finland
Oskari Heikinheimo	Hospital District of Helsinki and Uusimaa, Helsinki, Finland
Ilkka Kalliala	Hospital District of Helsinki and Uusimaa, Helsinki, Finland
Lauri Aaltonen	Hospital District of Helsinki and Uusimaa, Helsinki, Finland

Varpu Jokimaa	Hospital District of Southwest Finland, Turku, Finland
Johannes Kettunen	Northern Ostrobothnia Hospital District, Oulu, Finland
Marja Vääräsmäki	Northern Ostrobothnia Hospital District, Oulu, Finland
Outi Uimari	Northern Ostrobothnia Hospital District, Oulu, Finland
Laure Morin-Papunen	Northern Ostrobothnia Hospital District, Oulu, Finland
Maarit Niinimäki	Northern Ostrobothnia Hospital District, Oulu, Finland
Terhi Piltonen	Northern Ostrobothnia Hospital District, Oulu, Finland
Katja Kivinen	Institute for Molecular Medicine Finland (FIMM), HiLIFE, University of Helsinki, Helsinki, Finland
Elisabeth Widen	Institute for Molecular Medicine Finland (FIMM), HiLIFE, University of Helsinki, Helsinki, Finland
Taru Tukiainen	Institute for Molecular Medicine Finland (FIMM), HiLIFE, University of Helsinki, Helsinki, Finland
Mary Pat Reeve	Institute for Molecular Medicine Finland (FIMM), HiLIFE, University of Helsinki, Helsinki, Finland
Mark Daly	Institute for Molecular Medicine Finland (FIMM), HiLIFE, University of Helsinki, Helsinki, Finland; Broad Institute of MIT and Harvard; Massachusetts General Hospital
Niko Välimäki	University of Helsinki, Helsinki, Finland
Eija Laakkonen	University of Jyväskylä, Jyväskylä, Finland
Jaakko Tyrmi	University of Oulu, Oulu, Finland / University of Tampere, Tampere, Finland
Heidi Silven	University of Oulu, Oulu, Finland
Eeva Sliz	University of Oulu, Oulu, Finland
Riikka Arffman	University of Oulu, Oulu, Finland
Susanna Savukoski	University of Oulu, Oulu, Finland
Triin Laisk	Estonian biobank, Tartu, Estonia
Natalia Pujol	Estonian biobank, Tartu, Estonia
Mengzhen Liu	Abbvie, Chicago, IL, United States
Bridget Riley-Gillis	Abbvie, Chicago, IL, United States
Rion Pendergrass	Genentech, San Francisco, CA, United States
Janet Kumar	GlaxoSmithKline, Collegeville, PA, United States
Kirsi Auro	GlaxoSmithKline, Espoo, Finland
Iiris Hovatta	University of Helsinki, Finland
Chia-Yen Chen	Biogen, Cambridge, MA, United States
Erkki Isometsä	Hospital District of Helsinki and Uusimaa, Helsinki, Finland
Hanna Ollila	Institute for Molecular Medicine Finland (FIMM), HiLIFE, University of Helsinki, Helsinki, Finland
Jaana Suvisaari	Finnish Institute for Health and Welfare (THL), Helsinki, Finland
Thomas Damm Als	Aarhus University, Denmark
Antti Mäkitie	Department of Otorhinolaryngology - Head and Neck Surgery, University of Helsinki and Helsinki University Hospital, Helsinki, Finland
Argyro Bizaki-Vallaskangas	Pirkanmaa Hospital District, Tampere, Finland

Sanna Toppila-Salmi	University of Helsinki, Finland
Tytti Willberg	Hospital District of Southwest Finland, Turku, Finland
Elmo Saarentaus	Institute for Molecular Medicine Finland (FIMM), HiLIFE, University of Helsinki, Helsinki, Finland
Antti Aarnisalo	Hospital District of Helsinki and Uusimaa, Helsinki, Finland
Eveliina Salminen	Hospital District of Helsinki and Uusimaa, Helsinki, Finland
Elisa Rahikkala	Northern Ostrobothnia Hospital District, Oulu, Finland
Johannes Kettunen	Northern Ostrobothnia Hospital District, Oulu, Finland
Kristiina Aittomäki	Department of Medical Genetics, Helsinki University Central Hospital, Helsinki, Finland
Fredrik Åberg	Transplantation and Liver Surgery Clinic, Helsinki University Hospital, Helsinki University, Helsinki, Finland
Mitja Kurki	Institute for Molecular Medicine Finland (FIMM), HiLIFE, University of Helsinki, Helsinki, Finland; Broad Institute, Cambridge, MA, United States
Samuli Ripatti	Institute for Molecular Medicine Finland (FIMM), HiLIFE, University of Helsinki, Helsinki, Finland
Mark Daly	Institute for Molecular Medicine, Finland (FIMM), HiLIFE, University of Helsinki, Helsinki, Finland; Broad Institute of MIT and Harvard; Massachusetts General Hospital
Juha Karjalainen	Institute for Molecular Medicine Finland (FIMM), HiLIFE, University of Helsinki, Helsinki, Finland
Aki Havulinna	Institute for Molecular Medicine Finland (FIMM), HiLIFE, University of Helsinki, Helsinki, Finland; Finnish Institute for Health and Welfare (THL), Helsinki, Finland
Juha Mehtonen	Institute for Molecular Medicine Finland (FIMM), HiLIFE, University of Helsinki, Helsinki, Finland
Priit Palta	Institute for Molecular Medicine Finland (FIMM), HiLIFE, University of Helsinki, Helsinki, Finland
Shabbeer Hassan	Institute for Molecular Medicine Finland (FIMM), HiLIFE, University of Helsinki, Helsinki, Finland
Pietro Della Briotta Parolo	Institute for Molecular Medicine Finland (FIMM), HiLIFE, University of Helsinki, Helsinki, Finland
Wei Zhou	Broad Institute, Cambridge, MA, United States
Mutaamba Maasha	Broad Institute, Cambridge, MA, United States
Shabbeer Hassan	Institute for Molecular Medicine Finland (FIMM), HiLIFE, University of Helsinki, Helsinki, Finland
Susanna Lemmelä	Institute for Molecular Medicine Finland (FIMM), HiLIFE, University of Helsinki, Helsinki, Finland
Manuel Rivas	University of Stanford, Stanford, CA, United States
Mari E. Niemi	Institute for Molecular Medicine Finland (FIMM), HiLIFE, University of Helsinki, Helsinki, Finland
Aarno Palotie	Institute for Molecular Medicine Finland (FIMM), HiLIFE, University of Helsinki, Helsinki, Finland
Aoxing Liu	Institute for Molecular Medicine Finland (FIMM), HiLIFE, University of Helsinki, Helsinki, Finland
Arto Lehisto	Institute for Molecular Medicine Finland (FIMM), HiLIFE, University of Helsinki, Helsinki, Finland
Andrea Ganna	Institute for Molecular Medicine Finland (FIMM), HiLIFE, University of Helsinki, Helsinki, Finland

Vincent Llorens	Institute for Molecular Medicine Finland (FIMM), HiLIFE, University of Helsinki, Helsinki, Finland
Hannele Laivuori	Institute for Molecular Medicine Finland (FIMM), HiLIFE, University of Helsinki, Helsinki, Finland
Taru Tukiainen	Institute for Molecular Medicine Finland (FIMM), HiLIFE, University of Helsinki, Helsinki, Finland
Mary Pat Reeve	Institute for Molecular Medicine Finland (FIMM), HiLIFE, University of Helsinki, Helsinki, Finland
Henrike Heyne	Institute for Molecular Medicine Finland (FIMM), HiLIFE, University of Helsinki, Helsinki, Finland
Nina Mars	Institute for Molecular Medicine Finland (FIMM), HiLIFE, University of Helsinki, Helsinki, Finland
Joel Rämö	Institute for Molecular Medicine Finland (FIMM), HiLIFE, University of Helsinki, Helsinki, Finland
Elmo Saarentaus	Institute for Molecular Medicine Finland (FIMM), HiLIFE, University of Helsinki, Helsinki, Finland
Hanna Ollila	Institute for Molecular Medicine Finland (FIMM), HiLIFE, University of Helsinki, Helsinki, Finland
Rodos Rodosthenous	Institute for Molecular Medicine Finland (FIMM), HiLIFE, University of Helsinki, Helsinki, Finland
Satu Strausz	Institute for Molecular Medicine Finland (FIMM), HiLIFE, University of Helsinki, Helsinki, Finland
Tuula Palotie	University of Helsinki and Hospital District of Helsinki and Uusimaa, Helsinki, Finland
Kimmo Palin	University of Helsinki, Helsinki, Finland
Javier Garcia-Tabuenca	University of Tampere, Tampere, Finland
Harri Siirtola	University of Tampere, Tampere, Finland
Tuomo Kiiskinen	Institute for Molecular Medicine Finland (FIMM), HiLIFE, University of Helsinki, Helsinki, Finland
Jiwoo Lee	Institute for Molecular Medicine Finland (FIMM), HiLIFE, University of Helsinki, Helsinki, Finland; Broad Institute, Cambridge, MA, United States
Kristin Tsuo	Institute for Molecular Medicine Finland (FIMM), HiLIFE, University of Helsinki, Helsinki, Finland; Broad Institute, Cambridge, MA, United States
Amanda Elliott	Institute for Molecular Medicine Finland (FIMM), HiLIFE, University of Helsinki, Helsinki, Finland; Broad Institute, Cambridge, MA, USA and Massachusetts General Hospital, Boston, MA, USA
Kati Kristiansson	THL Biobank / Finnish Institute for Health and Welfare (THL), Helsinki, Finland
Mikko Arvas	Finnish Red Cross Blood Service / Finnish Hematology Registry and Clinical Biobank, Helsinki, Finland
Kati Hyvärinen	Finnish Red Cross Blood Service, Helsinki, Finland
Jarmo Ritari	Finnish Red Cross Blood Service, Helsinki, Finland
Olli Carpén	Helsinki Biobank / Helsinki University and Hospital District of Helsinki and Uusimaa, Helsinki
Johannes Kettunen	Northern Finland Biobank Borealis / University of Oulu / Northern Ostrobothnia Hospital District, Oulu, Finland
Katri Pylkäs	University of Oulu, Oulu, Finland
Eeva Sliz	University of Oulu, Oulu, Finland

Minna Karjalainen	University of Oulu, Oulu, Finland
Tuomo Mantere	Northern Finland Biobank Borealis / University of Oulu / Northern Ostrobothnia Hospital District, Oulu, Finland
Eeva Kangasniemi	Finnish Clinical Biobank Tampere / University of Tampere / Pirkanmaa Hospital District, Tampere, Finland
Sami Heikkinen	University of Eastern Finland, Kuopio, Finland
Arto Mannermaa	Biobank of Eastern Finland / University of Eastern Finland / Northern Savo Hospital District, Kuopio, Finland
Eija Laakkonen	University of Jyväskylä, Jyväskylä, Finland
Nina Pitkänen	Auria Biobank / University of Turku / Hospital District of Southwest Finland, Turku, Finland
Samuel Lessard	Translational Sciences, Sanofi R&D, Framingham, MA, USA
Clément Chatelain	Translational Sciences, Sanofi R&D, Framingham, MA, USA
Perttu Terho	Auria Biobank / University of Turku / Hospital District of Southwest Finland, Turku, Finland
Sirpa Soini	THL Biobank / Finnish Institute for Health and Welfare (THL), Helsinki, Finland
Jukka Partanen	Finnish Red Cross Blood Service / Finnish Hematology Registry and Clinical Biobank, Helsinki, Finland
Eero Punkka	Helsinki Biobank / Helsinki University and Hospital District of Helsinki and Uusimaa, Helsinki
Raisa Serpi	Northern Finland Biobank Borealis / University of Oulu / Northern Ostrobothnia Hospital District, Oulu, Finland
Sanna Siltanen	Finnish Clinical Biobank Tampere / University of Tampere / Pirkanmaa Hospital District, Tampere, Finland
Veli-Matti Kosma	Biobank of Eastern Finland / University of Eastern Finland / Northern Savo Hospital District, Kuopio, Finland
Teijo Kuopio	Central Finland Biobank / University of Jyväskylä / Central Finland Health Care District, Jyväskylä, Finland
Anu Jalanko	Institute for Molecular Medicine Finland (FIMM), HiLIFE, University of Helsinki, Helsinki, Finland
Huei-Yi Shen	Institute for Molecular Medicine Finland (FIMM), HiLIFE, University of Helsinki, Helsinki, Finland
Risto Kajanne	Institute for Molecular Medicine Finland (FIMM), HiLIFE, University of Helsinki, Helsinki, Finland
Mervi Aavikko	Institute for Molecular Medicine Finland (FIMM), HiLIFE, University of Helsinki, Helsinki, Finland
Henna Palin	Finnish Clinical Biobank Tampere / University of Tampere / Pirkanmaa Hospital District, Tampere, Finland
Malla-Maria Linna	Helsinki Biobank / Helsinki University and Hospital District of Helsinki and Uusimaa, Helsinki
Mitja Kurki	Institute for Molecular Medicine Finland (FIMM), HiLIFE, University of Helsinki, Helsinki, Finland; Broad Institute, Cambridge, MA, United States
Juha Karjalainen	Institute for Molecular Medicine Finland (FIMM), HiLIFE, University of Helsinki, Helsinki, Finland
Pietro Della Briotta Parolo	Institute for Molecular Medicine Finland (FIMM), HiLIFE, University of Helsinki, Helsinki, Finland
Arto Lehisto	Institute for Molecular Medicine Finland (FIMM), HiLIFE, University of Helsinki, Helsinki, Finland

Juha Mehtonen	Institute for Molecular Medicine Finland (FIMM), HiLIFE, University of Helsinki, Helsinki, Finland
Wei Zhou	Broad Institute, Cambridge, MA, United States
Masahiro Kanai	Broad Institute, Cambridge, MA, United States
Mutaamba Maasha	Broad Institute, Cambridge, MA, United States
Hannele Laivuori	Institute for Molecular Medicine Finland (FIMM), HiLIFE, University of Helsinki, Helsinki, Finland
Aki Havulinna	Institute for Molecular Medicine Finland (FIMM), HiLIFE, University of Helsinki, Helsinki, Finland; Finnish Institute for Health and Welfare (THL), Helsinki, Finland
Susanna Lemmelä	Institute for Molecular Medicine Finland (FIMM), HiLIFE, University of Helsinki, Helsinki, Finland
Tuomo Kiiskinen	Institute for Molecular Medicine Finland (FIMM), HiLIFE, University of Helsinki, Helsinki, Finland
L. Elisa Lahtela	Institute for Molecular Medicine Finland (FIMM), HiLIFE, University of Helsinki, Helsinki, Finland
Mari Kaunisto	Institute for Molecular Medicine Finland (FIMM), HiLIFE, University of Helsinki, Helsinki, Finland
Elina Kilpeläinen	Institute for Molecular Medicine Finland (FIMM), HiLIFE, University of Helsinki, Helsinki, Finland
Timo P. Sipilä	Institute for Molecular Medicine Finland (FIMM), HiLIFE, University of Helsinki, Helsinki, Finland
Oluwaseun Alexander Dada	Institute for Molecular Medicine Finland (FIMM), HiLIFE, University of Helsinki, Helsinki, Finland
Awaisa Ghazal	Institute for Molecular Medicine Finland (FIMM), HiLIFE, University of Helsinki, Helsinki, Finland
Anastasia Kytölä	Institute for Molecular Medicine Finland (FIMM), HiLIFE, University of Helsinki, Helsinki, Finland
Rigbe Weldatsadik	Institute for Molecular Medicine Finland (FIMM), HiLIFE, University of Helsinki, Helsinki, Finland
Sanni Ruotsalainen	Institute for Molecular Medicine Finland (FIMM), HiLIFE, University of Helsinki, Helsinki, Finland
Kati Donner	Institute for Molecular Medicine Finland (FIMM), HiLIFE, University of Helsinki, Helsinki, Finland
Timo P. Sipilä	Institute for Molecular Medicine Finland (FIMM), HiLIFE, University of Helsinki, Helsinki, Finland
Anu Loukola	Helsinki Biobank / Helsinki University and Hospital District of Helsinki and Uusimaa, Helsinki
Päivi Laiho	THL Biobank / Finnish Institute for Health and Welfare (THL), Helsinki, Finland
Tuuli Sistonen	THL Biobank / Finnish Institute for Health and Welfare (THL), Helsinki, Finland
Essi Kaiharju	THL Biobank / Finnish Institute for Health and Welfare (THL), Helsinki, Finland
Markku Laukkanen	THL Biobank / Finnish Institute for Health and Welfare (THL), Helsinki, Finland
Elina Järvensivu	THL Biobank / Finnish Institute for Health and Welfare (THL), Helsinki, Finland
Sini Lähteenmäki	THL Biobank / Finnish Institute for Health and Welfare (THL), Helsinki, Finland
Lotta Männikkö	THL Biobank / Finnish Institute for Health and Welfare (THL), Helsinki, Finland
Regis Wong	THL Biobank / Finnish Institute for Health and Welfare (THL), Helsinki, Finland
Auli Toivola	THL Biobank / Finnish Institute for Health and Welfare (THL), Helsinki, Finland
Minna Brunfeldt	THL Biobank / Finnish Institute for Health and Welfare (THL), Helsinki, Finland

Hannele Mattsson	THL Biobank / Finnish Institute for Health and Welfare (THL), Helsinki, Finland
Kati Kristiansson	THL Biobank / Finnish Institute for Health and Welfare (THL), Helsinki, Finland
Susanna Lemmelä	Institute for Molecular Medicine Finland (FIMM), HiLIFE, University of Helsinki, Helsinki, Finland
Sami Koskelainen	THL Biobank / Finnish Institute for Health and Welfare (THL), Helsinki, Finland
Tero Hiekkalinna	THL Biobank / Finnish Institute for Health and Welfare (THL), Helsinki, Finland
Teemu Paajanen	THL Biobank / Finnish Institute for Health and Welfare (THL), Helsinki, Finland
Priit Palta	Institute for Molecular Medicine Finland (FIMM), HiLIFE, University of Helsinki, Helsinki, Finland
Kalle Pärn	Institute for Molecular Medicine Finland (FIMM), HiLIFE, University of Helsinki, Helsinki, Finland
Mart Kals	Institute for Molecular Medicine Finland (FIMM), HiLIFE, University of Helsinki, Helsinki, Finland
Shuang Luo	Institute for Molecular Medicine Finland (FIMM), HiLIFE, University of Helsinki, Helsinki, Finland
Tarja Laitinen	Pirkanmaa Hospital District, Tampere, Finland
Mary Pat Reeve	Institute for Molecular Medicine Finland (FIMM), HiLIFE, University of Helsinki, Helsinki, Finland
Shanmukha Sampath Padmanabhuni	Institute for Molecular Medicine Finland (FIMM), HiLIFE, University of Helsinki, Helsinki, Finland
Marianna Niemi	University of Tampere, Tampere, Finland
Harri Siirtola	University of Tampere, Tampere, Finland
Javier Gracia-Tabuenca	University of Tampere, Tampere, Finland
Mika Helminen	University of Tampere, Tampere, Finland
Tiina Luukkaala	University of Tampere, Tampere, Finland
Iida Vähätalo	University of Tampere, Tampere, Finland
Jyrki Pitkänen	Institute for Molecular Medicine Finland (FIMM), HiLIFE, University of Helsinki, Helsinki, Finland
Marco Hautalahti	Finnish Biobank Cooperative - FINBB
Johanna Mäkelä	Finnish Biobank Cooperative - FINBB
Sarah Smith	Finnish Biobank Cooperative - FINBB
Tom Southerington	Finnish Biobank Cooperative - FINBB

Supplementary References

1 Ajeawung, N. F. et al. Mutations in ANAPC1, Encoding a Scaffold Subunit of the Anaphase-Promoting Complex, Cause Rothmund-Thomson Syndrome Type 1. American journal of human genetics 105, 625-630, doi:10.1016/j.ajhg.2019.06.011 (2019).

2 Demetriou, M., Binkert, C., Sukhu, B., Tenenbaum, H. C. & Dennis, J. W. Fetuin/alpha2-HS glycoprotein is a transforming growth factor-beta type II receptor mimic and cytokine antagonist. The Journal of biological chemistry 271, 12755-12761, doi:10.1074/jbc.271.22.12755 (1996).

3 Szweras, M. et al. alpha 2-HS glycoprotein/fetuin, a transforming growth factor-beta/bone morphogenetic protein antagonist, regulates postnatal bone growth and remodeling. The Journal of biological chemistry 277, 19991-19997, doi:10.1074/jbc.M112234200 (2002).

4 Schinke, T. et al. The serum protein alpha2-HS glycoprotein/fetuin inhibits apatite formation in vitro and in mineralizing calvaria cells. A possible role in mineralization and calcium homeostasis. The Journal of biological chemistry 271, 20789-20796, doi:10.1074/jbc.271.34.20789 (1996).

5 Heiss, A. et al. Structural basis of calcification inhibition by alpha 2-HS glycoprotein/fetuin-A. Formation of colloidal calciprotein particles. The Journal of biological chemistry 278, 13333-13341, doi:10.1074/jbc.M210868200 (2003).

6 Price, P. A. & Lim, J. E. The inhibition of calcium phosphate precipitation by fetuin is accompanied by the formation of a fetuin-mineral complex. The Journal of biological chemistry 278, 22144-22152, doi:10.1074/jbc.M300744200 (2003).

7 Rittenberg, B. et al. Regulation of BMP-induced ectopic bone formation by Ahsg. J. Orthop. Res. 23, 653-662, doi:10.1016/j.orthres.2004.11.010 (2005).

8 Gowen, L. C. et al. Targeted disruption of the osteoblast/osteocyte factor 45 gene (OF45) results in increased bone formation and bone mass. J. Biol. Chem. 278, 1998-2007, doi:10.1074/jbc.M203250200 (2003).

9 Gullard, A. et al. MEPE Localization in the Craniofacial Complex and Function in Tooth Dentin Formation. J. Histochem. Cytochem. 64, 224-236, doi:10.1369/0022155416635569 (2016).

10 Staines, K. A. et al. MEPE is a novel regulator of growth plate cartilage mineralization. Bone 51, 418-430, doi:10.1016/j.bone.2012.06.022 (2012).

11 Schrauwen, I. et al. Variants affecting diverse domains of MEPE are associated with two distinct bone disorders, a craniofacial bone defect and otosclerosis. Genetics in medicine : official journal of the American College of Medical Genetics 21, 1199-1208, doi:10.1038/s41436-018-0300-5 (2019).

12 Malaval, L. et al. Bone sialoprotein plays a functional role in bone formation and osteoclastogenesis. J Exp Med 205, 1145-1153, doi:10.1084/jem.20071294 (2008).

13 Wu, M., Chen, G. & Li, Y.-P. TGF-β and BMP signaling in osteoblast, skeletal development, and bone formation, homeostasis and disease. Bone Research 4, 16009, doi:10.1038/boneres.2016.9 (2016).

14 Crane, J. L. & Cao, X. Bone marrow mesenchymal stem cells and TGF-β signaling in bone remodeling. J Clin Invest 124, 466-472, doi:10.1172/JCI70050 (2014).

15 Komori, T. Regulation of Proliferation, Differentiation and Functions of Osteoblasts by Runx2. International journal of molecular sciences 20, doi:10.3390/ijms20071694 (2019).

16 Barutcu, A. R. et al. The bone-specific Runx2-P1 promoter displays conserved three-dimensional chromatin structure with the syntenic Supt3h promoter. Nucleic Acids Res. 42, 10360-10372, doi:10.1093/nar/gku712 (2014).

17 Moffatt, P. et al. Metaphyseal dysplasia with maxillary hypoplasia and brachydactyly is caused by a duplication in RUNX2. Am. J. Hum. Genet. 92, 252-258, doi:10.1016/j.ajhg.2012.12.001 (2013).

18 Komori, T. et al. Targeted disruption of Cbfa1 results in a complete lack of bone formation owing to maturational arrest of osteoblasts. Cell 89, 755-764, doi:10.1016/s0092-8674(00)80258-5 (1997).

19 Moffatt, P. et al. Metaphyseal dysplasia with maxillary hypoplasia and brachydactyly is caused by a duplication in RUNX2. Am. J. Hum. Genet. 92, 252-258, doi:10.1016/j.ajhg.2012.12.001 (2013).

20 Chang, J. L. et al. Tissue-specific calibration of extracellular matrix material properties by transforming growth factor-β and Runx2 in bone is required for hearing. EMBO Rep 11, 765-771, doi:10.1038/embor.2010.135 (2010).

21 Bizet, A. A. et al. CD109-mediated degradation of TGF-β receptors and inhibition of TGF-β responses involve regulation of SMAD7 and Smurf2 localization and function. Journal of Cellular Biochemistry 113, 238-246, doi:https://doi.org/10.1002/jcb.23349 (2012).

22 Nakajima, M. et al. A genome-wide association study identifies susceptibility loci for ossification of the posterior longitudinal ligament of the spine. Nat. Genet. 46, 1012-1016, doi:10.1038/ng.3045 (2014).

23 Negishi-Koga, T. et al. Suppression of bone formation by osteoclastic expression of semaphorin 4D. Nat Med 17, 1473-1480, doi:10.1038/nm.2489 (2011).

24 Robertson, I. B. et al. Latent TGF-β-binding proteins. Matrix biology : journal of the International Society for Matrix Biology 47, 44-53, doi:10.1016/j.matbio.2015.05.005 (2015).

25 McInerney-Leo, A. M. et al. Mutations in LTBP3 cause acromicric dysplasia and geleophysic dysplasia. J. Med. Genet. 53, 457-464, doi:10.1136/jmedgenet-2015-103647 (2016).

26 Dugan, S. L. et al. New recessive truncating mutation in LTBP3 in a family with oligodontia, short stature, and mitral valve prolapse. Am. J. Med. Genet. A 167, 1396-1399, doi:10.1002/ajmg.a.37049 (2015).

27 McInerney-Leo, A. M. et al. Mutations in LTBP3 cause acromicric dysplasia and geleophysic dysplasia. J. Med. Genet. 53, 457-464, doi:10.1136/jmedgenet-2015-103647 (2016).

28 Huckert, M. et al. Mutations in the latent TGF-beta binding protein 3 (LTBP3) gene cause brachyolmia with amelogenesis imperfecta. Hum. Mol. Genet. 24, 3038-3049, doi:10.1093/hmg/ddv053 (2015).

29 Dabovic, B. et al. Osteopetrosis-like phenotype in latent TGF-beta binding protein 3 deficient mice. Bone 37, 25-31, doi:10.1016/j.bone.2005.02.021 (2005).

30 Puente, X. S. et al. Exome sequencing and functional analysis identifies BANF1 mutation as the cause of a hereditary progeroid syndrome. American journal of human genetics 88, 650-656, doi:10.1016/j.ajhg.2011.04.010 (2011).

31 Guerrini, M. M. et al. Human osteoclast-poor osteopetrosis with hypogammaglobulinemia due to TNFRSF11A (RANK) mutations. American journal of human genetics 83, 64-76, doi:10.1016/j.ajhg.2008.06.015 (2008).

32 Calabrese, G. M. et al. Integrating GWAS and Co-expression Network Data Identifies Bone Mineral Density Genes SPTBN1 and MARK3 and an Osteoblast Functional Module. Cell Systems 4, 46-59.e44, doi:https://doi.org/10.1016/j.cels.2016.10.014 (2017).

33 Chang, E. J. et al. Brain-type creatine kinase has a crucial role in osteoclast-mediated bone resorption. Nat. Med. 14, 966-972, doi:10.1038/nm.1860 (2008).

34 Nakao, A. et al. TGF-beta receptor-mediated signalling through Smad2, Smad3 and Smad4. Embo j 16, 5353-5362, doi:10.1093/emboj/16.17.5353 (1997).

35 Chen, X., Zhang, K., Hock, J., Wang, C. & Yu, X. Enhanced but hypofunctional osteoclastogenesis in an autosomal dominant osteopetrosis type II case carrying a c.1856C>T mutation in CLCN7. Bone Research 4, 16035, doi:10.1038/boneres.2016.35 (2016).

36 Waguespack, S. G. et al. Chloride channel 7 (ClCN7) gene mutations and autosomal dominant osteopetrosis, type II. J. Bone Miner. Res. 18, 1513-1518, doi:10.1359/jbmr.2003.18.8.1513 (2003).

37 Pangrazio, A. et al. Molecular and clinical heterogeneity in CLCN7-dependent osteopetrosis: report of 20 novel mutations. Hum. Mutat. 31, E1071-1080, doi:10.1002/humu.21167 (2010).

38 Cleiren, E. et al. Albers-Schönberg disease (autosomal dominant osteopetrosis, type II) results from mutations in the ClCN7 chloride channel gene. Human molecular genetics 10, 2861-2867, doi:10.1093/hmg/10.25.2861 (2001).

39 Moosa, S. et al. Novel compound heterozygous mutations in TELO2 in a patient with severe expression of You-Hoover-Fong syndrome. Mol Genet Genomic Med 5, 580-584, doi:10.1002/mgg3.287 (2017).

40 Bonnard, C. et al. Mutations in IRX5 impair craniofacial development and germ cell migration via SDF1. Nat Genet 44, 709-713, doi:10.1038/ng.2259 (2012).

41 Cain, C. J. et al. Loss of Iroquois homeobox transcription factors 3 and 5 in osteoblasts disrupts cranial mineralization. Bone Rep 5, 86-95, doi:10.1016/j.bonr.2016.02.005 (2016).

42 O'Sullivan, J. et al. Whole-Exome sequencing identifies FAM20A mutations as a cause of amelogenesis imperfecta and gingival hyperplasia syndrome. American journal of human genetics 88, 616-620, doi:10.1016/j.ajhg.2011.04.005 (2011).

43 de la Dure-Molla, M. et al. Pathognomonic oral profile of Enamel Renal Syndrome (ERS) caused by recessive FAM20A mutations. Orphanet J Rare Dis 9, 84, doi:10.1186/1750-1172-9-84 (2014).

44 Kinoshita, A. et al. Domain-specific mutations in TGFB1 result in Camurati-Engelmann disease. Nat Genet 26, 19-20, doi:10.1038/79128 (2000).

45 Clements, M. E. & Johnson, R. W. PREX1 drives spontaneous bone dissemination of ER+ breast cancer cells. Oncogene 39, 1318-1334, doi:10.1038/s41388-019-1064-3 (2020).

46 Bowl, M. R. et al. A large scale hearing loss screen reveals an extensive unexplored genetic landscape for auditory dysfunction. Nat Commun 8, 886-886, doi:10.1038/s41467-017-00595-4 (2017).

47 Dickinson, M. E. et al. High-throughput discovery of novel developmental phenotypes. Nature 537, 508-514, doi:10.1038/nature19356 (2016).

48 Zhang, T., Xu, J. & Xu, P.-X. Eya2 expression during mouse embryonic development revealed by Eya2(lacZ) knockin reporter and homozygous mice show mild hearing loss. Dev Dyn 250, 1450-1462, doi:10.1002/dvdy.326 (2021).
