# Supplementary material for: Genome-wide screen of otosclerosis in population biobanks: 27 loci and shared associations with skeletal structure
Source: Nat Commun. 2023 Jan 18;14:157. doi: 10.1038/s41467-022-32936-3 (PMC9849444; doi:10.1038/s41467-022-32936-3)
Supplement: Supplementary file 2 — Description of Additional Supplementary Files [file 41467_2022_32936_MOESM2_ESM.pdf]

Description of Additional Supplementary Files

File Name: Supplementary Data 1
Description: **Case and control characteristics**. Gender and baseline age are reported for cases and controls in each study cohort.

File Name: Supplementary Data 2
Description: **Lead variants in FinnGen.** Lead variants are presented for the GWAS in FinnGen (including 1,563 cases and 249,281 controls), annotated with effect estimates, effect allele frequencies and *p*-values in the other two cohorts. GWAS was performed using a generalized mixed model with the saddlepoint approximation using SAIGE v0.20, using a kinship matrix as a random effect and covariates as fixed effects. A Bonferroni-corrected two-sided genome-wide *p*-value threshold of 5×10^{-8} was used to account for multiple comparisons. *Chr = chromosome, EA = effect allele, EAF = effect allele frequency, NEA = non-effect allele, OR = odds ratio, CI = confidence interval.*

File Name: Supplementary Data 3
Description: **Lead variants in Estonian Biobank**. Lead variants are presented for the GWAS in Estonian Biobank (including 985 cases and 196,516 controls), annotated with effect estimates, effect allele frequencies and *p*-values in the other two cohorts. GWAS was performed using a generalized mixed model with the saddlepoint approximation using SAIGE v0.20, using a kinship matrix as a random effect and covariates as fixed effects. A Bonferroni-corrected two-sided genome-wide *p*-value threshold of 5×10^{-8} was used to account for multiple comparisons. *Chr = chromosome, EA = effect allele, EAF = effect allele frequency, NEA = non-effect allele, OR = odds ratio, CI = confidence interval.*

File Name: Supplementary Data 4
Description: **Lead variants in UK Biobank.** Lead variants are presented for the GWAS in UK Biobank (including 956 cases and 415,401 controls), annotated with effect estimates, effect allele frequencies and *p*-values in the other two cohorts. GWAS was performed using a generalized mixed model with the saddlepoint approximation using SAIGE v0.20, using a kinship matrix as a random effect and covariates as fixed effects. A Bonferroni-corrected two-sided genome-wide *p*-value threshold of 5×10^{-8} was used to account for multiple comparisons. *Chr = chromosome, EA = effect allele, EAF = effect allele frequency, NEA = non-effect allele, OR = odds ratio, CI = confidence interval.*

File Name: Supplementary Data 5
Description: **SNP-based heritability of otosclerosis in each cohort.** Approximate estimates for the narrow sense heritability of otosclerosis in each cohort and the subsequent meta-analysis were obtained and compared using three different summary statistics based methods: 1) LD Score Regression (LDSC), and 2) BLK-LDAK and 3) LDAK-Thin of the SumHer software. We used summary statistics separately from the meta-analysis and each cohort, restricting the analyses to variants present in HapMap3. For the BLK-LDAK and LDAK-Thin models, we used the European tagging files based on UKBB (http://dougspeed.com/pre-computed-tagging-files/), and for LDSC we used LD Scores computed using 1000 Genomes European data (https://data.broadinstitute.org/alkesgroup/LDSCORE/eur_w_ld_chr.tar.bz2). For the liability transformations, a population prevalence approximation of 0.3% was used.

File Name: Supplementary Data 6

Description: **LD score regression intercepts and attenuation ratios for each cohort.** Lambda GC, mean χ^2, intercepts and attenuation ratios [(LD score regression intercept – 1) / (mean χ2 – 1)] from LD score regression are presented separately for each cohort.

File Name: Supplementary Data 7
Description: **Lead variants from the meta-analysis with cohort-level estimates.** Lead variants from the meta-analysis (including a total of 3,504 cases and 861,198 controls) are presented, additionally annotated with cohort-specific effect allele frequencies, cohort-specific *p*-values and heterogeneity statistics. GWAS for each individual study cohort was performed using a generalized mixed model with the saddlepoint approximation using SAIGE v0.20, using a kinship matrix as a random effect and covariates as fixed effects, and results were combined with a fixed-effect meta-analysis. A Bonferroni-corrected two-sided genome-wide *p*-value threshold of $5x10^{-8}$ was used to account for multiple comparisons. *Chr = chromosome, EA = effect allele, EAF = effect allele frequency, NEA = non-effect allele, OR = odds ratio, CI = confidence interval.*

File Name: Supplementary Data 8
Description: **Lead variants from suggestive level association loci.** *Chr = chromosome, EA = effect allele, EAF = effect allele frequency, NEA = non-effect allele, OR = odds ratio, CI = confidence interval.* GWAS for each individual study cohort was performed using a generalized mixed model with the saddlepoint approximation using SAIGE v0.20, using a kinship matrix as a random effect and covariates as fixed effects, and results were combined with a fixed-effect meta-analysis including a total of 3,504 cases and 861,198 controls. Lead variants are shown for loci which reached a *p*-value < $1x10^{-6}$ in the meta-analysis but were not significant at the genome-wide multiple comparison corrected threshold of $5x10^{-8}$.

File Name: Supplementary Data 9
Description: **Significantly associated missense variants in the meta-analysis.** Missense variants which were significantly associated with otosclerosis ($p < 5x10^{-8}$) in the meta-analysis are shown. GWAS for each individual study cohort was performed using a generalized mixed model with the saddlepoint approximation using SAIGE v0.20, using a kinship matrix as a random effect and covariates as fixed effects, and results were combined with a fixed-effect meta-analysis including a total of 3,504 cases and 861,198 controls. A Bonferroni-corrected two-sided genome-wide *p*-value threshold of $5x10^{-8}$ was used to account for multiple comparisons. The Ensembl Variant Effect Predictor tool was used for variant annotation.

File Name: Supplementary Data 10
Description: **Missense variants in high LD with lead variants from the meta-analysis.** Six missense variants were in high LD ($r^2 > 0.6$) with the lead variants from the meta-analysis based on linkage disequilibrium in the FinnGen cohort. GWAS for each individual study cohort was performed using a generalized mixed model with the saddlepoint approximation using SAIGE v0.20, using a kinship matrix as a random effect and covariates as fixed effects, and results were combined with a fixed-effect meta-analysis including a total of 3,504 cases and 861,198 controls. A Bonferroni-corrected two-sided genome-wide *p*-value threshold of $5x10^{-8}$ was used to account for multiple comparisons. The Ensembl Variant Effect Predictor tool was used for variant annotation.

File Name: Supplementary Data 11
Description: **Summary of fine-mapping results in FinnGen.** GWAS in was performed using a generalized mixed model with the saddlepoint approximation using SAIGE v0.20, using a kinship matrix as a random effect and covariates as fixed effects. All regions in which the lead variant reached a multiple

comparison corrected p-value of < 1x10-6 in FinnGen were fine-mapped using SuSiE. A 3Mb window (± 1.5Mb) was used around each lead variant, and LD was calculated between variants from individual-level FinnGen data. *CS = credible set, PIP = posterior inclusion probability.*

File Name: Supplementary Data 12
Description: **Variant-level fine-mapping results in FinnGen.** Variant-level results are shown for good-quality credible sets. GWAS in was performed using a generalized mixed model with the saddlepoint approximation using SAIGE v0.20, using a kinship matrix as a random effect and covariates as fixed effects. All regions in which the lead variant reached a multiple comparison corrected p-value of < 1x10-6 in FinnGen were fine-mapped using SuSiE. A 3Mb window (± 1.5Mb) was used around each lead variant, and LD was calculated between variants from individual-level FinnGen data.

File Name: Supplementary Data 13
Description: **Phenome-wide association study based on GWAS Atlas (summary).** GWAS for each individual study cohort was performed using a generalized mixed model with the saddlepoint approximation using SAIGE v0.20, using a kinship matrix as a random effect and covariates as fixed effects, and results were combined with a fixed-effect meta-analysis including a total of 3,504 cases and 861,198 controls. A Bonferroni-corrected two-sided genome-wide *p*-value threshold of $5x10^{-8}$ was used to account for multiple comparisons. For all lead variants from 27 significantly associated loci, we queried GWAS Atlas (a comprehensive database of publicly available GWAS summary statistics) for significant associations among 4,756 reported traits. Traits which were significantly associated with the lead variants at the Bonferroni-adjusted threshold of $p < 1.05x10^{-5}$ (to account for the number of tested traits) are shown.

File Name: Supplementary Data 14
Description: **Directional concordance or discordance between otosclerosis and recurring traits in the phenome-wide association study based on GWAS Atlas.** GWAS for each individual study cohort was performed using a generalized mixed model with the saddlepoint approximation using SAIGE v0.20, using a kinship matrix as a random effect and covariates as fixed effects, and results were combined with a fixed-effect meta-analysis including a total of 3,504 cases and 861,198 controls. A Bonferroni-corrected two-sided genome-wide *p*-value threshold of $5x10^{-8}$ was used to account for multiple comparisons. For all lead variants from 27 significantly associated loci, we queried GWAS Atlas (a comprehensive database of publicly available GWAS summary statistics) for significant associations among 4,756 reported traits. Traits which were significantly associated with the lead variants at the Bonferroni-adjusted threshold of $p < 1.05x10^{-5}$ (to account for the number of tested traits) are shown.

File Name: Supplementary Data 15
Description: **Directional concordance or discordance between otosclerosis and recurring traits in the phenome-wide association study based on GWAS Atlas.** When all reported associations reaching nominal significance (*p*-value < 0.05) in GWAS Atlas were included, seven traits were associated with at least ten otosclerosis GWAS lead variants. The presence of significant directional concordance or discordance of effect estimates for each trait and otosclerosis was evaluated using a two-tailed binomial test. A Bonferroni-corrected *p*-value threshold of 0.05/7 was used to evaluate statistical significance.

File Name: Supplementary Data 16
Description: **Phenome-wide association study for rs753138805 in FinnGen.** For the *MEPE* frameshift variant rs753138805 observable only in FinnGen and not included in GWAS Atlas, we performed a phenome-wide association analysis of 2,861 registry-based disease endpoints in FinnGen using a

generalized mixed model with the saddlepoint approximation using SAIGE v0.20, using a kinship matrix as a random effect and covariates as fixed effects. Results for all nominally significant associations ($p<0.05$) are shown, and significant associations at the Bonferroni-corrected threshold of 0.05/2,861 are highlighted in bold. *MAF = minor allele frequency.*

File Name: Supplementary Data 17
Description: **Genetic correlations based on LDHub.** We tested the genetic correlations of otosclerosis with all available bone, anthropometric and lung traits (54 traits in total) in LDHub, a centralized database of summary-level GWAS results. Genetic correlation analyses were conducted using the automated pipeline after filtering the meta-analysis summary statistics to the HapMap3 variants recommended by the authors (v1.9.3.; http://ldsc.broadinstitute.org/ldhub/).[1-3] A Bonferroni-corrected two-sided *p*-value threshold ($\alpha = 0.05/54$) was used to evaluate statistical significance.

File Name: Supplementary Data 18
Description: **Previous GWAS reports for all significant variants from the meta-analysis.** Using the GWAS Catalog database (accessed on 22 April 2020), previous GWAS results were queried for all 1,257 variants which were significantly associated with otosclerosis ($p < 5x10^{-8}$) in the meta-analysis. *GWAS = genome-wide association study.*

File Name: Supplementary Data 19
Description: **Significant genes in a gene-based analysis with MAGMA.** The MAGMA v1.08 software was used to identify genes associated with otosclerosis based on effect estimates from the meta-analysis. Variants were mapped to 18,877 genes based on their RefSNP numbers. A gene-based analysis was performed using the default SNPwise-mean model and a Bonferroni-corrected two-sided p-value threshold to account for the number of tested genes ($\alpha = 0.05/18,877$).

File Name: Supplementary Data 20
Description: **GO Biological Process enrichment for the protein-coding genes nearest to lead variants.** A GO Biological Process (BP) enrichment analysis was performed for the 27 genes nearest to the lead variants from the meta-analysis. The analysis was performed using the online tool provided by the Gene Ontology Consortium connected to the PANTHER classification system (http://geneontology.org/docs/go-enrichment-analysis/ and http://pantherdb.org). Enriched biological processes with a False Discovery Rate (FDR) corrected two-sided *p*-value under 0.05 are reported.

File Name: Supplementary Data 21
Description: **Summary of colocalization with eQTL catalogue.** Results from a colocalization analysis integrating otosclerosis GWAS and eQTL data are shown. Expression data from the EMBL-EBI eQTL catalogue for 24 tissues/cell types were fine-mapped previously by Alasoo and Keriamov. For each gene, we calculated the causal posterior probability (CLPP) and causal posterior agreement (CLPA) as detailed in the Methods section. Based on a causal posterior probability cutoff of ≥ 0.1%, we report genes whose expression colocalizes genetically with otosclerosis in at least one tissue. The analyses were not corrected for multiple comparisons. For each gene, the number of studies and tissues exceeding this cutoff are shown, as well as the highest observed CLPP and CLPA.

File Name: Supplementary Data 22
Description: **Colocalization with gene expression based on the eQTL Catalogue.** All colocalization results with a causal posterior probability (CLPP) exceeding 0.1% are presented. *CLPA* = causal posterior agreement.

File Name: Supplementary Data 23
Description: **Replication of candidate gene variants.** Meta-analysis effect estimates and *p*-values are shown for variants previously reported to be associated with otosclerosis. References to the original studies are presented in the main text.

File Name: Supplementary Data 24
Description: **Lead variants from FinnGen annotated with results from additional GWAS analyses.** Lead variants from the primary FinnGen GWAS (including a total of 1,563 cases and 249,281 controls) are annotated with odds ratios and *p*-values from additional GWAS analyses in FinnGen. Additional GWAS were conducted in FinnGen for women (1002 cases and 140,222 controls) and men (561 cases and 109,059 controls). GWAS were performed using a generalized mixed model with the saddlepoint approximation using SAIGE v0.20, using a kinship matrix as a random effect and covariates as fixed effects. A Bonferroni-corrected two-sided genome-wide *p*-value threshold of $5x10^{-8}$ was used to account for multiple comparisons.

File Name: Supplementary Data 25
Description: **Lead variants from a female-only GWAS in FinnGen.** GWAS analyses in FinnGen were performed for women only (1002 cases and 140,222 controls) and men only (561 cases and 109,059 controls). GWAS were performed using a generalized mixed model with the saddlepoint approximation using SAIGE v0.20, using a kinship matrix as a random effect and covariates as fixed effects. A Bonferroni-corrected two-sided genome-wide *p*-value threshold of $5x10^{-8}$ was used to account for multiple comparisons.Seven lead variants from loci reaching genome-wide significance ($p < 5x10^{-8}$) are shown, annotated with corresponding odds ratios and *p*-values from the male-only GWAS.

File Name: Supplementary Data 26
Description: **Lead variants from a male-only GWAS in FinnGen.** GWAS analyses in FinnGen were performed for men only (561 cases and 109,059 controls) and women only (1002 cases and 140,222 controls). GWAS were performed using a generalized mixed model with the saddlepoint approximation using SAIGE v0.20, using a kinship matrix as a random effect and covariates as fixed effects. A Bonferroni-corrected two-sided genome-wide *p*-value threshold of $5x10^{-8}$ was used to account for multiple comparisons. Four lead variants from loci reaching genome-wide significance ($p < 5x10^{-8}$) are shown, annotated with corresponding odds ratios and *p*-values from the female-only GWAS.
