# Supplementary material for: Genome-wide screen of otosclerosis in population biobanks: 27 loci and shared associations with skeletal structure
Source: Nat Commun. 2023 Jan 18;14:157. doi: 10.1038/s41467-022-32936-3 (PMC9849444; doi:10.1038/s41467-022-32936-3)
Supplement: Supplementary file 4 — Reporting Summary [file 41467_2022_32936_MOESM4_ESM.pdf]

nature portfolio

Corresponding author(s): Aarno Palotie

Last updated by author(s): Jul 18, 2022

Reporting Summary

Nature Portfolio wishes to improve the reproducibility of the work that we publish. This form provides structure for consistency and transparency in reporting. For further information on Nature Portfolio policies, see our Editorial Policies and the Editorial Policy Checklist.

Statistics

For all statistical analyses, confirm that the following items are present in the figure legend, table legend, main text, or Methods section.

n/a	Confirmed	
☐	☒	The exact sample size (*n*) for each experimental group/condition, given as a discrete number and unit of measurement
☐	☒	A statement on whether measurements were taken from distinct samples or whether the same sample was measured repeatedly
☐	☒	The statistical test(s) used AND whether they are one- or two-sided<br>*Only common tests should be described solely by name; describe more complex techniques in the Methods section.*
☐	☒	A description of all covariates tested
☐	☒	A description of any assumptions or corrections, such as tests of normality and adjustment for multiple comparisons
☐	☒	A full description of the statistical parameters including central tendency (e.g. means) or other basic estimates (e.g. regression coefficient) AND variation (e.g. standard deviation) or associated estimates of uncertainty (e.g. confidence intervals)
☐	☒	For null hypothesis testing, the test statistic (e.g. *F*, *t*, *r*) with confidence intervals, effect sizes, degrees of freedom and *P* value noted<br>*Give P values as exact values whenever suitable.*
☐	☒	For Bayesian analysis, information on the choice of priors and Markov chain Monte Carlo settings
☐	☒	For hierarchical and complex designs, identification of the appropriate level for tests and full reporting of outcomes
☐	☒	Estimates of effect sizes (e.g. Cohen's *d*, Pearson's *r*), indicating how they were calculated

Our web collection on statistics for biologists contains articles on many of the points above.

Software and code

Policy information about availability of computer code

Data collection	No software was used was data collection
Data analysis	GenomeStudio v2.0.4, Eagle v2.3, SAIGE v0.20, LiftOver v1.12, GWAMA v2.2.2, LDSC v1.0.1, SumHer (LDAK v5.0), PLINK v1.07, SUSiE v1.0, MAGMA v1.08, Locuszoom.js and primer3 v1.1.4. Selected analyses were restricted to variants included in the HapMap3 reference panel.

For manuscripts utilizing custom algorithms or software that are central to the research but not yet described in published literature, software must be made available to editors and reviewers. We strongly encourage code deposition in a community repository (e.g. GitHub). See the Nature Portfolio guidelines for submitting code & software for further information.

Data

Policy information about availability of data

All manuscripts must include a data availability statement. This statement should provide the following information, where applicable:

- Accession codes, unique identifiers, or web links for publicly available datasets
- A description of any restrictions on data availability
- For clinical datasets or third party data, please ensure that the statement adheres to our policy

The meta-analysis summary statistics generated in this study have been deposited in the NHGRI-EBI GWAS Catalog under the accession code GCST90129575. Cohort-level summary statistics from FinnGen are publicly available (https://r6.finngen.fi/pheno/H8_OTOSCLE). Individual-level genotypes and register data from FinnGen participants can be accessed by approved researchers via the Fingenious portal (https://site.fingenious.fi/en/) hosted by the Finnish Biobank Cooperative FinBB (https://finbb.fi/en/). Data from the UK Biobank are available by application to all bone fine researchers in the public interest. The individual-level Estonian Biobank data are available under restricted access administered by Estonian Genome Center of the University of Tartu (EGCUT) in accordance with the regulations of the Estonian Human Genes Research Act; access can be obtained by application at www.biobank.ee.

We used the following publicly available datasets for downstream analyses of genome-wide association study results: HapMap3 (https://www.sanger.ac.uk/resources/downloads/human/hapmap3.html), Ensembl Variant Effect Predictor (https://useast.ensembl.org/info/docs/tools/vep/index.html), GWAS Atlas database (https://atlas.ctglab.nl), LDHub (http://ldsc.broadinstitute.org/ldhub/), GWAS Catalog (https://www.ebi.ac.uk/gwas/), ClinVar (https://www.ncbi.nlm.nih.gov/clinvar), Molecular Signature Database v7.1 (http://www.gsea-msigdb.org/gsea/msigdb), the Gene Ontology Consortium connected to the PANTHER classification system (http://geneontology.org/docs/go-enrichment-analysis/ and http://pantherdb.org), EMBL-EBI eQTL Catalogue (https://www.ebi.ac.uk/eqtl/), and International Mouse Phenotyping Consortium (IMPC) data (https://www.mousephenotype.org).

Field-specific reporting

Please select the one below that is the best fit for your research. If you are not sure, read the appropriate sections before making your selection.

☒ Life sciences ☐ Behavioural & social sciences ☐ Ecological, evolutionary & environmental sciences

For a reference copy of the document with all sections, see nature.com/documents/nr-reporting-summary-flat.pdf

Life sciences study design

All studies must disclose on these points even when the disclosure is negative.

Sample size	All available otosclerosis cases were identified across the available biobanks, resulting in 3,504 otosclerosis cases and 861,198 controls. The study was considered well powered as the sample size was many times larger than the sample size of a previous otosclerosis GWAS (Schrauwen et al, 2009) which had identified one genome-wide association locus.
Data exclusions	Individuals were excluded from genomic analyses following standard genotype quality control protocols, e.g. if their call-rate was < 95% or if their sex defined based on heterozygosity of the X chromosome did not match the sex in their registered phenotype data.
Replication	Genome-wide association analyses of otosclerosis were conducted in all three biobanks and the results were meta-analyzed. Since individual-level genotype data could not be merged due to data privacy laws, selected analyses based on individual-level data (linkage disequilibrium estimation and fine-mapping) were performed in the cohort with the largest amount of cases (FinnGen).
Randomization	No randomization occurred. Participant age, participant sex, genomic principal components (1-10) and genotyping batches were used as fixed-effect covariates in genome-wide association analyses with SAIGE v0.20.
Blinding	No intervention occurred and blinding was not necessary.

Reporting for specific materials, systems and methods

We require information from authors about some types of materials, experimental systems and methods used in many studies. Here, indicate whether each material, system or method listed is relevant to your study. If you are not sure if a list item applies to your research, read the appropriate section before selecting a response.

Materials & experimental systems

n/a	Involved in the study	
☐	☒	Antibodies
☒	☐	Eukaryotic cell lines
☒	☐	Palaeontology and archaeology
☐	☒	Animals and other organisms
☐	☒	Human research participants
☒	☐	Clinical data
☒	☐	Dual use research of concern

Methods

n/a	Involved in the study	
☒	☐	ChIP-seq
☒	☐	Flow cytometry
☒	☐	MRI-based neuroimaging

Antibodies

Antibodies used	Rabbit polyclonal anti-MEPE (Kerafast, Boston, MA, #ENH086-FP, 1:400;); rabbit monoclonal RUNX2 (Cell Signaling Technology, Danvers, MA, clone D1L7F, #12556, 1:200); goat anti-rabbit Alexa Fluor 488 antibodies (Thermo Fisher Scientific, Waltham, MA, #A-11008, 1:500); goat anti-rabbit IgG (Abcam, Cambridge, UK, #ab64256, 5 µg/mL)
Validation	Validation of anti-MEPE antibody: Gluhak-Heinrich, J., Pavlin, D., Yang, W., MacDougall, M. & Harris, S. E. MEPE expression in osteocytes during orthodontic tooth movement. Arch Oral Biol 52, 684-690, doi:10.1016/j.archoralbio.2006.12.010 (2007).

Animals and other organisms

Policy information about studies involving animals; ARRIVE guidelines recommended for reporting animal research

Laboratory animals	Pregnant CD1 mice were obtained from Charles River Laboratories, Wilmington, CA, and were housed at 20-22°C ambient

Laboratory animals	temperature with 30-70% humidity, a 12-hour light/dark cycle, and food and water available ad libitum. Postnatal day 2 (P2) mice, postnatal day 6 (P6) mice, postnatal day 12 (P12) mice and adult, 3 month old mice of both sexes were studied.
Wild animals	No wild animals were used in the study.
Field-collected samples	No field collected samples were used in the study.
Ethics oversight	All procedures were approved by the Administrative Panel on Laboratory Animal Care (APLAC) at Stanford University according to the National Institutes of Health guidelines for animal care (protocol #33998).

Note that full information on the approval of the study protocol must also be provided in the manuscript.

Human research participants

Policy information about studies involving human research participants

Population characteristics	We identified individuals with ICD-based otosclerosis diagnoses from three national biobank-based cohorts: The Finnish FinnGen cohort, the Estonian Biobank (EstBB) and the UK Biobank (UKBB). Written informed consent was obtained from all study participants at recruitment. Cases were 35.9% male with mean age of 60.3 (SD 13.4) in FinnGen, 22.5% male with mean age of 54.3 (SD 14.9) in EstBB and 40.5% male with mean age of 59.0 (SD 7.14) in UKBB. Controls were 43.7% male with mean age of 52.8 (SD 17.6) in FinnGen, 34.5% men with mean age of 44.1 (SD 16.2) in EstBB and 45.9% men with mean age of 56.8 (SD 8.0) in UKBB.
Recruitment	Data comprised in FinnGen Data Freeze 6 are administered by regional biobanks (Auria Biobank, Biobank of Central Finland, Biobank of Eastern Finland, Borealis Biobank, Helsinki Biobank, Tampere Biobank), the Blood Service Biobank, the Terveystalo Biobank, and biobanks administered by the Finnish Institute for Health and Welfare. EstBB is a population-based cohort of 200,000 participants with a rich variety of phenotypic and health-related information collected for each individual. At recruitment, participants have signed a consent to allow follow-up linkage of their electronic health records (EHR), thereby providing a longitudinal collection of phenotypic information. UKBB comprises phenotype data from 500,000 volunteer participants from the UK population aged between 40 and 69 years during recruitment in 2006-2010. Data for all participants have been linked with national Hospital Episode Statistics.
Ethics oversight	The FinnGen study protocol (HUS/990/2017) was approved by the Ethics Review Board of the Hospital District of Helsinki and Uusimaa. The Estonian Biobank study has obtained approval from the Ethics Review Committee on Human Research of the University of Tartu. UK Biobank has approval from the North West Multi-centre Research Ethics Committee (MREC) as a Research Tissue Bank (RTB) approval. All animal procedures followed the approved institutional protocol at Stanford University according to the National Institutes of Health guidelines for animal care.

Note that full information on the approval of the study protocol must also be provided in the manuscript.
